# Cheminformatics Bioprospection of Broad Spectrum Plant Secondary Metabolites Targeting the Spike Proteins of Omicron Variant and Wild-Type SARS-CoV-2

**DOI:** 10.3390/metabo12100982

**Published:** 2022-10-17

**Authors:** Jamiu Olaseni Aribisala, Christiana Eleojo Aruwa, Taofik Olatunde Uthman, Ismaila Olanrewaju Nurain, Kehinde Idowu, Saheed Sabiu

**Affiliations:** 1 Department of Biotechnology and Food Science, Faculty of Applied Science, Durban University of Technology, P.O. Box 1334, Durban 4000, South Africa; 2 Department of Biochemistry, Faculty of Natural and Applied Sciences, Nile University of Nigeria, Abuja FCT 900001, Nigeria; 3 Department of Applied Health Sciences, Faculty of Science and Mathematics, Brock University, St. Catharines, ON L2S 3A1, Canada

**Keywords:** SARS-CoV-2, omicron, spike protein, corn silk, *Crescentia cujete*, molecular docking, molecular dynamic simulation, COVID-19

## Abstract

The spike protein (SP) of SARS-CoV-2 (SC-2) is susceptible to high mutation and has contributed to the multiple waves of COVID-19 being experienced. Hence, targeting the SP remains a logical approach in the development of potent therapeutics against SARS-CoV-2. Here, a computational technique was adopted to identify broad-spectrum plant secondary metabolites with indigenous relevance in the management of respiratory infections against the SPs of the SC-2 wild- type (SC-2WT) and omicron variants. Following 100 ns molecular dynamic (MD) simulation and binding free energy calculation of the top five compounds identified through molecular docking, maysin (SC-2WT (−34.85 kcal/mol), omicron (−38.88 kcal/mol)) and geraniin (SC-2WT (−36.90 kcal/mol) omicron (−31.28 kcal/mol)) had better broad-spectrum activities for the investigated SPs than zafirlukast (SC-2WT (−33.73 kcal/mol) omicron (−22.38 kcal/mol)). Furthermore, 6-hydroxycyanidin-3-rutinoside (−42.97 kcal/mol) and kaempferol-7-glucoside (−37.11 kcal/mol) had the best affinity for the SPs of omicron and SC-2WT, respectively. Interestingly, except for Kaempferol-7-glucoside against omicron SP, all the top-ranked compounds were thermodynamically stable with the SP of both variants, and this observation was linked to the number, nature, and bond length in the resulting complexes in each case. Also, except for geraniin, all the top-ranked compounds had lower toxicity profiles compared to zafirlukast and this could be attributed to their phenolic moieties. Nevertheless, the in vitro and in vivo confirmation of the activities observed in this study is recommended, especially for maysin and geraniin with the best broad-spectrum activity, towards development of COVID-19 drug candidates.

## 1. Introduction

Coronavirus disease (COVID-19) is a respiratory disease caused by the SARS-CoV-2 (SC-2), a virus in the family Coronaviridae [1]. SARS-CoV-2 infection was first reported in China in December 2019, and was later classified as pandemic by the World Health Organization (WHO) in March 2020 [2]. The disease spread to about 218 countries and has resulted in over 6.5 million deaths as of 16 September 2022 [3,4,5]. SARS-CoV-2 is a 30 kbp RNA virus that encodes four structural proteins and sixteen non-structural proteins [6]. Several of these proteins have been identified as possible targets to reduce pathogenesis [5,6]. The spike protein (SP), nucleocapsid, envelope, and membrane protein are the four main structural proteins of SC-2 [1]. However, the SP remains the most crucial element in viral transmission and pathogenesis as it helps in receptor binding and membrane fusion [7]. Although, most of the population worldwide has been vaccinated, reports suggest a possible resurgence of COVID-19 infection due to SC-2 genetic modification [8]. Omicron (B.1.1.529), a novel SC-2 variant that emerged in South Africa in 2021, is highly transmissible with over 100,000 of its genomes having evolved and dominated SC-2 infections around the world by early 2022 [8]. The variant is more transmissible than previous strains (such as Delta (B.1.617.2), Delta (AY.4.2), Beta (B.1.351), and Alpha (B.1.1.7)), possibly due to its higher number of mutations [7]. These mutations have been primarily identified in the SP [7,8]; hence, the need for effective therapeutics targeting the SP.

Given the repeated waves of COVID-19 [8], and the fact that none of the currently administered vaccines has had 100% effectiveness in curbing SC-2 infections caused by all the variants [7], there is a need to develop specialized drugs for COVID-19 infection. All the drugs currently approved by the Food and Drug Administration (FDA) for COVID-19 treatment, such as remedesivir and olumiant, as well those authorised for use under emergency use authorization (EUA), such as bebtelovimab and lagevrio, were repurposed for use with several conditions attached to their usage to limit side effects [3]. Thus, findings indicating that conventional medicine has been used successfully to treat COVID-19 [9] have opened the door to alternative therapies. Among the alternative therapeutic options, the use of phytochemicals to combat viral infections is well documented [9,10]. This study forms part of the on-going efforts at developing potent therapeutics against the druggable targets of SC-2, and adopted a computational approach to screen metabolites from corn silk, an underutilized waste product of corn cultivation [11], and *Crescentia cujete*, an economical and medicinal plant with wide indigenous uses against several ailments including respiratory diseases [12,13], against SP of the SARS-CoV-2 wild-type (SC-2WT) and omicron variants to identify probable broad spectrum leads. Phytoconstituents previously identified as promising anti-SC-2 metabolites [14] were also included to make the library of compounds considered in this study. Given the high relevance of SP in the spread of COVID-19 infection, screening bioactives from plants against the target could identify potential compounds that could be further developed as COVID-19 therapeutics.

## 2. Materials and Methods

### 2.1. Library of Plant Secondary Metabolites

A total of 73 compounds (Supplementary Table S1), 58 of which had been reported to have been isolated from different parts of *C. cujete* [13,15], 14 phytoconstituents from corn silk [11] and some of those previously identified as promising anti-SC-2 metabolites [14] were used in this study and will be regarded as LOCM going forward.

### 2.2. Collection and Preparation of LOCM and Reference Standards

The 3D structures of the ligands {LOCM and those of the reference standard (zafirlukast [16], cefoperazone [17]), were obtained from PubChem “https://pubchem.ncbi.nlm.nih.gov/ (accessed on 15 January 2022)” and subsequently optimized via the addition of Gasteiger charges on UCSF chimera software V1.14 [18] in preparation for docking. Zafirlukast cefoperazone are good, repurposed drug candidates for COVID-19 targeting SARS-CoV-2 SP [16,17] and hence their selection as the reference standards in this study.

### 2.3. Collection and Preparation of SC-2WT and Omicron SPs

The X-ray crystal structures of SP (SC-2WT (6LZG) and omicron (7T9J)) were obtained from the Protein Data Bank “https://www.rcsb.orgs/ (accessed on 17 January 2022)”and optimized as earlier reported [19] using UCSF chimera software V1.14 [18] in readiness for molecular docking.

### 2.4. SC-2WT and Omicron SP Active Sites Identification and Molecular Docking of Ligands

The x-y-z coordinates of the receptor-binding domain (RBD) of SC-2WT (centre (x: −40.96, y: 43.37, z: 0.39); size (x: 20.80, y: 20.90, z: 20.94)) and omicron SP (centre (x: 158.98, y: 204.78, z: 243.60); size (x: 27.99, y: 20.78, z: 26.55)) were determined using Discovery Studio version 21.1.0 [20] and afterward validated from the literature [21,22]. Selecting the amino acid at the RBD of each target and fitting the lattice box to the predefined x-y-z coordinates ensured subsequent docking to the active sites of the proteins [23]. Docking of the optimized 3D structures of the ligands and proteins was done using the Autodock vina module (although other scoring functions are available, Autodock vina has been reported for its high hit rate for COVID-19 studies [24]) on Python Prescription (PyRx) v 0.9.5, which permits multiple docking of ligands [25]. Finally, the ligands were sorted according to their interactions and affinity, and the best pose docked complexes with the highest affinity for the top five compounds were retrieved and subjected to MD simulations.

### 2.5. Docking Protocol Validation

**Figure 1:**
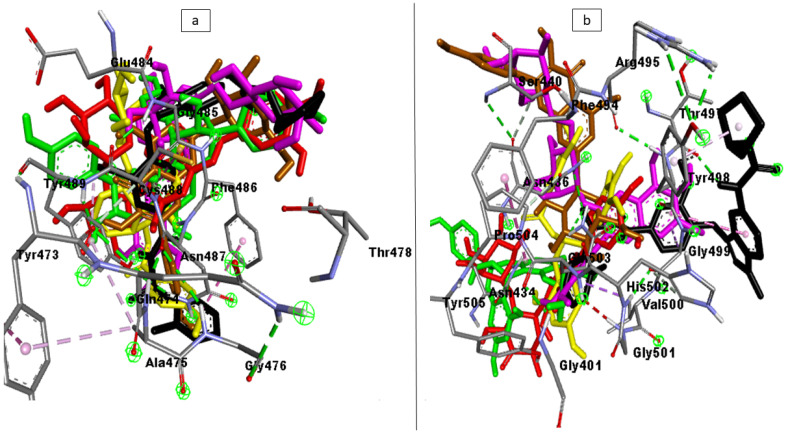
The superimposition and interactions of the top-ranked investigated LOCM compounds (Maysin (red), 6-Hydroxylcyanidin-3-rutinoside (purple), Kaempferol-7-glucoside (brown), Geraniin (green), epigallocatechin gallate (yellow), and cataposide (yellow)), and reference standard (black), with the highest binding affinity with the RBD residues of the co-crystal structure of (**a**) SC-2WT and (**b**) Omicron SP.

The docking studies were validated by analyzing the ligand interactions with SC-2WT (6LZG) and omicron (7T9J) SPs’ receptor-binding domain (RBD) for Angiotensin-Converting Enzyme 2 (ACE2). The most energy-minimized conformation (highest binding free energy) for each of the ligands was observed to interact with higher numbers of bonds and nature of bonds including hydrogen interactions with the RBD residues of both SC-2WT SP (489, 486, 484, 487, 475, 473, and 478) [21] and omicron SP (353, 496, 505, 501, 500, 498, and 493) [22] relative to other poses. The superimposition of the top-ranked compounds and zafirlukast (standard with the highest docking score) had an RMSD value of <1 Å from each other which suggests preferred docking position for all the compounds on the SP of both targets. Also, the RBD amino acid interactions of the top-ranked compound as well as zafirlukast with SC-2WT SP, which include residues 489 and 484, and omicron SP, such as residues 495 and 498, correlate with those reported by Vardhan and Sahoo [7]. This observation demonstrates that the docking scores observed for the compounds and the standards are a result of their interactions with the RBD co-crystal structure of the SPs and hence validates the docking protocol used. The superimposition and interactions of the top five compounds and the zafirlukast are presented in Figure 1.

### 2.6. Molecular Dynamics Simulation

The MD simulation was performed as previously described [25]. In short, the simulations were run over a 100-ns period using the AMBER 14 package. The FF18SB variant of the AMBER force field was adopted to describe the operating systems. The ANTECHAMBER was used to generate the atomic partial charges of the ligands by exploiting General Amber Force Field (GAFF) and Restrained Electrostatic Potential (RESP) methods. The hydrogen atoms, Na^+^, and Cl^−^ counter ions of the Leap module were used to neutralize the systems. In each case of the simulation, the amino acid residues were numbered accordingly, and the systems were suspended in an orthorhombic box of TIP3P water molecules such that all atoms were within 8 Å of each box edge. For each simulation, an initial minimization of 2000 steps was performed using a restraint potential of 500 kcal/mol. They were carried out for 990 steps with the steepest descent technique, followed by 990 steps with conjugate grades. The conjugate gradient approach was used to perform an additional 990 steps of complete minimization (without restraint). Heating MD simulations from 0 K to 300 K (gradual) were run for 50 ps, with the systems maintaining a constant number of atoms and volume. The solutes in the systems have a potential harmonic constraint of 10 kcal/mol and a collision frequency of 1.0 ps. Following that, each system was equilibrated for approximately 500 ps, with the operating temperatures held constant at 300 K [25]. The MD simulation was run for 100 ns and in each case, the bonds of hydrogen atoms were constrained using the SHAKE algorithm. Each MD simulation had a step size of 2 fs, which corresponded to the isobarisothermic ensemble (NPT) with randomized seeding, a temperature of 300 K, a constant pressure of 1 bar, a Langevin thermostat with a collision frequency of 1.0 ps, and a pressure-coupling constant of 2 ps. The results of the 100 ns MD simulation were regarded as post-dynamic data.

### 2.7. Post-MD Simulation

The post-MD simulation was performed as previously reported [19]. Following the simulation, the coordinates of the systems were merged and evaluated using the AMBER 14 PTRAJ module. Thereafter, the CPPTRAJ module was used to assess the root mean square fluctuation (RMSF), the radius of gyration (RoG), root mean square deviation (RMSD), and solvent accessibility surface area (SASA), and raw data were obtained using Origin V6. Similarly, using the Molecular Mechanics/GB Surface Area technique, the free binding energy (G) in each simulation instance was computed by averaging 100,000 snapshots from a 100 ns MD simulation trajectory using the expression G_bind_ = G_complex_ − (G_Receptor_ + G_ligand_). The interactions of the complexes in each simulation were visualized using Discovery Studio version 21.1.0, which adopted a MdmDiscoveryScript to provide functions, properties, constants, and data structures.

### 2.8. Pharmacokinetic Analysis and Molecular Fingerprinting of the Top-Ranked LOCM

In this study, the SwissADME web “http://swissadme.ch/index.php (accessed on 15 March 2022)” and Molinspiration “https://www.molinspiration.com/cgi-bin/properties (accessed on 15 March 2022)” toolkits were used to predict the physicochemical properties, pharmacokinetics, drug-likeness, and medicinal chemistry friendliness of LOCM, while the toxicological profiles were evaluated using the Protox II webserver “https://tox-new.charite.de/protox_II/ (accessed on 15 March 2022)” [14]. All the top ranked investigated compounds were molecularly fingerprinted using Galaxy Europe “https://usegalaxy.eu./# (accessed on 8 June 2022)”. In short, Galaxy Europe’s “molecule-to-fingerprint” program was used to convert the chemical smile format into “pen Babel FP2 fingerprints”. The “Open Babel FP2 fingerprints” were then clustered using the Taylor-Butina and NxN clustering fingerprinting techniques with thresholds of 0.8 and 0.0, respectively [15].

## 3. Results and Discussion

Molecular docking is a structure-based virtual screening technique that ranks compounds based on their orientations and interactions at a target-binding site, with a higher negative value indicating a better affinity for the target [26]. Using this technique, the LOCM compounds were ranked against the RBD of SC-2WT and omicron SPs. The LOCM docking scores against SC-2WT SP range from −2.4 kcal/mol to 8.4 kcal/mol, with phosphorous acid and maysin having the lowest and highest docking scores, respectively (Supplementary Table S1). At least 12 and 2 compounds from LOCM had higher docking scores against SC-2WT SP than cefoperazone (−6.7 kcal/mol) and zafirlukast (−7.9 kcal/mol), respectively (Supplementary Table S1). The identification of LOCM compounds with higher docking scores than the two reference standards indicates their wide range affinities for the SP of SC-2WT. This observation is not surprising, as some of the compounds have been previously shown to have anti-SC-2 properties [14]. However, as a result of the multiple mutations in the RBD of omicron SP [8], identifying compounds with affinities towards the target will be remarkable. Interestingly, after sorting, LOCM had docking scores ranging from −2.6 to −7.5 kcal/mol (Supplementary Table S1), with at least 21 and 2 compounds having higher docking scores than cefoperazone (−6.2 kcal/mol) and zafirlukast (−7.4 kcal/mol), respectively. Notably, among the top-scoring LOCM compounds, four compounds (maysin, geraniin, Kaempferol-7-glucoside, and 6-hydroxycyanidin-3) had better broad-spectrum affinity toward the SPs of SC-2WT and omicron than cefoperazone (Table 1). On the other hand, compared to zafirlukast, only maysin had better broad-spectrum activity against both targets (Table 1). The better broad-spectrum of activities than cefoperazone observed with four of the compounds suggest their better potential as omicron and SC-2WT SP inhibitors relative to cefoperazone. Similarly, maysin having better broad-spectrum activity than both reference standards suggests its better advantage over other LOCM compounds as an anti-COVID-19 drug candidate. However, since molecular docking study only provides preliminary assessment of the interactions of compounds with a target without considering the dynamics of the protein, further MD simulations were performed on the top-ranked compounds to understand their thermodynamic compatibility with the targets.

The thermodynamic binding free energy measures the difference in energy between a complex and its unbound component, and the higher the negative value, the better the ligand’s affinity for the protein [24]. Since zafirlukast among the reference standards demonstrated better affinity towards the SPs of omicron and SC-2WT through molecular docking, zafirlukast was selected as the standard for thermodynamic binding free energy calculation and subsequent MD simulation analysis. Remarkably, two (maysin, and 6-hydroxycyanidin-3) of the four LOCM compounds with broad-spectrum activities against both variants had better affinity toward the SP of omicron than SC-2WT (Table 2). Maysin and 6-hydroxycyanidin-3, with binding free energies of −34.85 kcal/mol and −28.84 kcal/mol, respectively, against SC-2WT SP had −38.88 kcal/mol and −42.97 kcal/mol against omicron SP (Table 2). The better affinity of maysin and 6-hydroxycyanidin-3 against omicron SP suggests their likely capability to modulate the mutated RBD of omicron than geraniin and Kaempferol-7-glucoside and hence their advantage as omicron SP inhibitors. However, of the four compounds with broad spectrum activities identified through the molecular docking, only maysin (SC-2WT (−34.85 kcal/mol), omicron (−38.88 kcal/mol) and geraniin (SC-2WT (−36.90 kcal/mol), omicron (−31.28 kcal/mol)) had better binding free energy against SPs of both variants than zafirlukast (SC-2WT/omicron: −33.73 kcal/mol/−22.38 kcal/mol) after 100 ns MD simulation (Table 2). While this observation partially agrees with the results of the molecular docking, it further highlights the superior broad-spectrum advantage of maysin and geraniin as omicron and SC-2WT SPs inhibitors relative to zafirlukast. Specifically, against both targets, 6-Hydroxycyanidin 3-rutinoside and Kaempferol-7-glucoside had the highest binding free energy against the omicron and SC-2WT SPs, respectively (Table 2). Generally, while maysin and 6-hydroxycyanidin-3 had higher binding free energy towards the SPs of omicron than SC-2WT, geraniin and Kaempferol-7-glucoside, on the other hand, had better affinity towards SC-2WT than omicron SP. Remarkably, maysin and geraniin showed superior broad-spectrum advantage against SP of both variants than zafirlukast, while 6-Hydroxycyanidin 3-rutinoside and Kaempferol-7-glucoside had the best binding free energy against the SPs of omicron and SC-2WT, respectively. All these observations point to the different capabilities of the top-ranked compounds, with maysin and geraniin being the best in terms of their broad-spectrum capability to act against infection that may arise as a result of either the SC-2WT or omicron variant.

**Figure 2:**
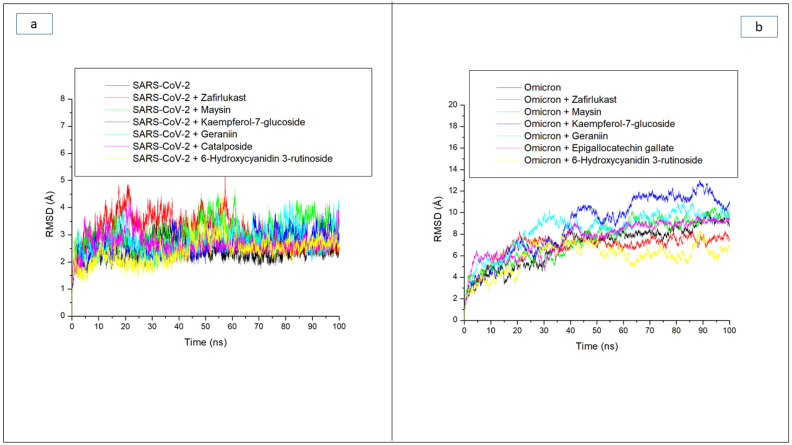
Comparative root mean squared deviation (RMSD) plots of alpha-carbon, top five LOCM compounds, and zafirlukast against the spike protein of (**a**) SARS-CoV-2 wild-type and (**b**) omicron, after 100 ns MD simulation period.

The RMSD trajectory shows the time-dependent deviation of a complex structure from its apo structure, and the lower the RMSD value, the closer the complex structure is to the apo structure, which signifies stability [27]. Following equilibrating of the atoms of each of the systems during the first 10 ns of the simulation, the ligand-SP complexes in each case of the two targets, as well as their apo-SPs, had major swaying that was more prominent in the ligand-omicron SP complexes relative to the ligand complexes with SC-2WT SP (Figure 2a,b). Consequently, complexes formed with the omicron SP generally had higher RMSD values relative to those formed with SC-2WT SP. Except for maysin against SP of SC-2WT, with an RMSD value of 3.07 Å, all the other top-ranked compounds had RMSD values that were less than 3 Å (Figure 2a, Table 3), suggestive of good structural stability and compatibility with SC-2WT spike protein. Interestingly, all the top five compounds had lower RMSD values than zafirlukast (3.15 Å) (Figure 2a, Table 3), which could be an indication of their better potential in promoting the structural stability of SC-2WT SPs and as potent inhibitors. This observation is partially in accordance with the docking study where geraniin and Kaempferol-7-glucoside showed better affinity towards SC-2WT SP than zafirlukast. However, compared to the apo-SC-2WT SP, ligand binding generally increased RMSD values (Figure 2a, Table 3), which agrees with an earlier study [24], where the binding of verbascoside to SARS-CoV-2 SP caused increased RMSD value. As depicted in the RMSD plot, higher swaying was more prominent in the ligand-omicron SP complexes during the 100 ns simulation (Figure 2b), which was more noticeable in the systems between 20 ns and 100 ns. Of the investigated compounds, Kaempferol-7-glucoside complexed with omicron with an average RMSD value of 9.44 Å had the highest fluctuation, especially between 20 ns and 90 ns when the system fluctuated from 4 Å to about >12 Å (Figure 2b). Similarly, the geraniin-omicron SP complex with an average RMSD value of 8.71 Å also had a high fluctuation from 4.5 Å at 15 ns to 10 Å after 80 ns (Figure 2b)**.** The high fluctuation observed with Kaempferol-7-glucoside and geraniin against omicron SP in this study could be suggestive of their lesser stability with the protein and hence a disadvantage in their capability to inhibit the protein. Kaempferol-7-glucoside showing lesser stability for the omicron SP is not surprising as the compound had the least binding free energy towards the protein and geraniin, though had promising affinity towards omicron SP, and displayed better affinity with SC-2WT SP. The other investigated compounds as well as zafirlukast though had major fluctuation during the simulation (Figure 2b) and average values that were higher than 3 Å (Table 3), a limit for good deviation [26], generally promoting the internal stability of omicron spike protein. This observation is apparent as 6-Hydroxycyanidin 3-rutinoside (5.94 Å), epigallocatechin gallate (7.60 Å), maysin (8.25 Å), and zafirlukast (6.99 Å) when complexed with omicron SP had RMSD values that were lesser than the unbound omicron SP (8.56 Å), with 6-Hydroxycyanidin 3-rutinoside having the lowest value (Table 3). The 6-Hydroxycyanidin 3-rutinoside having the lowest RMSD value signifying its better stability for omicron SP further corroborates the binding free energy study where the compound had the best affinity for omicron SP. Generally, among the two compounds (geraniin and maysin) with better broad-spectrum activity than zafirlukast, maysin seems to be more thermodynamically compatible with omicron SP than SC-2WT SP and vice versa for geraniin. Similarly, 6-Hydroxycyanidin 3-rutinoside and Kaempferol-7-glucoside, with the best affinity towards omicron SP and SC-2WT SP, respectively, also had the lowest RMSD value against the respective protein, which further supports their advantage as potential inhibitors of the respective targets.

**Figure 3:**
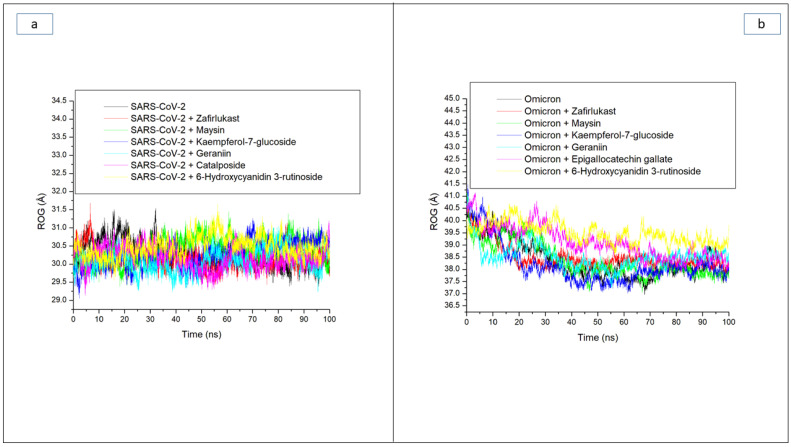
Comparative radius of gyration (ROG) plots of alpha-carbon, top five LOCM compounds, and zafirlukast against the spike protein of (**a**) SARS-CoV-2 wild-type and (**b**) omicron, after 100 ns MD simulation period.

Besides RMSD, the ROG also measures stability and the overall compactness of a ligand–receptor complex, and its lower values connote better complex folding with less conformational entropy [28]. In this study, fewer fluctuations in compactness observed with the SC-2WT SP system (Figure 3a) during the 100 ns simulation period could mean that the binding of the investigated high-ranked compounds and zafirlukast to the SC-2WT SP induced little effect on its folding. This observation was supported by their respective average ROG values with an overall small variation of 0.3 Å among all systems, with the geraniin-SC-2WT SP complex having the lowest values (Table 3). A logical inference from this observation is that, though top-ranked compounds do not cause excessive folding of the SC-2WT SP, they do however maintain the thermodynamic entropy of the protein, and this is consistent with the results of RMSD values, suggesting their thermodynamic stability towards SC-2WT SP. However, against the omicron SP, a continuous reduction in ROG value depicts folding was observed throughout the 100 ns simulation period for all the systems (Figure 3b). This observation was more prominent with the unbound omicron SP (38.07 Å) relative to the bound systems (Table 3), which could be an indication that the increased structural stability in terms of RMSD observed with the investigated compounds and zafirlukast against the omicron SP relative to the unbound omicron SP was not due to protein folding. This observation is consistent with the findings of Aribisala and Sabiu [23], where the structural stability observed in the RMSD finding of phenolics and penicillin-binding protein 2a contradicted ROG predictions. Among the bound systems, Kaempferol-7-glucoside (38.12 Å) followed by maysin (38.20 Å) had the lowest average ROG values against omicron SP (Table 3). Kaempferol-7-glucoside having the lowest average ROG value against omicron SP is surprising, as the compound had the lowest binding free energy with the highest RMSD value against the target. Similarly, 6-Hydroxycyanidin 3-rutinoside (39.50 Å), with the highest average ROG value, had the highest binding free energy and lowest average RMSD value against omicron SP (Table 3). These observations regarding the average ROG values further imply that the increased structural compatibility noted with the average RMSD values in this study was not due to protein folding, and further corroborate earlier findings that ROG may be affected by several characteristics specific to the protein such as protein topology, size, length, point of interactions, and amino acid packing [24,29,30]. In the case of omicron spike protein, the multiple mutations in the target could have affected the protein topology, size, and length relative to SC-2 SP and hence the observed variations in the average RMSD and ROG values of both targets. Between the two compounds (geraniin and maysin) with a better broad spectrum of activity than zafirlukast, maysin seems to be more thermodynamically compatible with omicron SP than SC-2WT SP and vice versa for geraniin, further corroborating the RMSD findings. On the other hand, 6-Hydroxycyanidin 3-rutinoside and Kaempferol-7-glucoside, with the best affinity and structural stability towards omicron SP and SC-2WT SP, respectively, had the highest average ROG values against the respective protein, which further emphasized that the increased structural stability observed with the compounds might not be due to protein folding.

**Figure 4:**
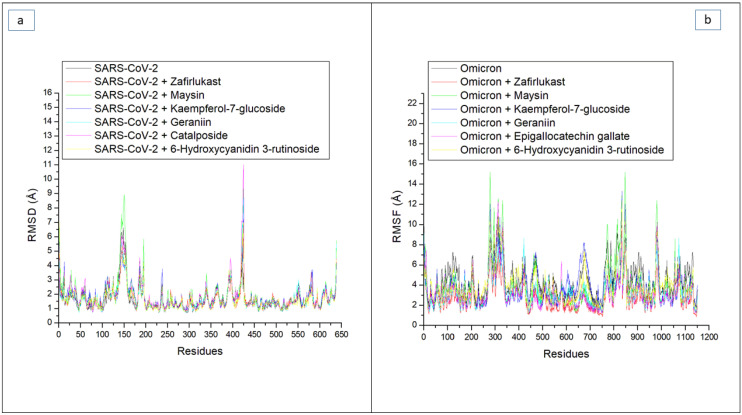
Comparative root means squares fluctuation (RMSF) plots of alpha-carbon, top five LOCM compounds, and zafirlukast against the spike protein of (**a**) SARS-CoV-2 wild-type and (**b**) omicron, after 100 ns MD simulation period.

By measuring the RMSF value, the thermodynamic flexibility of amino acid residues at the RBD of both SPs was investigated. The lower the RMSF value, the better the stability of the intra- and intermolecular bonds formed [31]. In this study, a high fluctuation in the RMSF plot was observed in the SC-2WT SP systems between residues 125 and 175 and residues 400 and 425. Specifically, the RBD residues of SC-2WT SP (489, 486, 484, 487, 475, 473, and 478) [21] did not fall into the region of high swaying (Figure 4a), suggesting their active involvement in intra- and intermolecular binding within the protein, hence contributing to the structural stability observed in the protein. Consequently, higher RMSF values were observed in the whole protein relative to the RBD region of the SC-2WT SP in all the systems (Table 3 and Table 4). The lowest RMSF value in the whole protein (1.64 Å) (Table 3) and at the RBD region (1.13 Å) (Table 4) was noted in the unbound SC-2WT SP and thus depicts that binding generally causes increased flexibility of SC-2WT SP amino acid residues. This observation is in tandem with the report of Mousavi et al. [32] who also observed significant fluctuation of the RBD residues of SC-2 SP following binding with some Iranian medicinal plant phytoconstituents. Among the bound systems, the 6-Hydroxycyanidin 3-rutinoside (1.66 Å) had the lowest RMSF value, which suggests its higher capability to reduce the flexibility of SC-2WT SP amino acid residues. However, in the RBD, geraniin (1.20 Å) had the lowest RMSF value (Table 3 and Table 4). Generally, the RMSF values in the whole protein and at the RBD of SC-2WT SP were within the acceptable limit (less than 3 Å), which could be suggestive of good intra- and intermolecular binding in the SC-2WT SP systems. Unlike the SC-2WT SP plot, the residues in all the systems in the omicron SP plot had higher fluctuations throughout the simulation period (Figure 4b). This was more prominent in residues between 250 and 350, and 750 and 1150 (Figure 4b) where there was major swaying, and less prominent in residues between 400 and 425, 450 and 500, and 650 and 700 with minimal swaying. Specifically, none of the RBD residues (353, 496, 505, 501, 500, 498, and 493) [22] falls in the region of major swaying and only four (493, 496, 498, and 500) fall within the region of minor swaying (Figure 4b). These observations could have been the reason for the observed lesser average RMSF values at the RBD of omicron SP relative to the whole protein in all the systems (Table 3 and Table 4). As with the SC-2WT systems, lesser fluctuation in the RBD of omicron SP relative to the whole proteins in all the systems could suggest the active contribution of this region in the intra- and intermolecular binding and hence an advantage in the inhibition of the protein. Unlike the SAR-CoV-2 systems, the highest RMSF value in the whole protein (4.42 Å) and at the RBD (3.33 Å) was noted in the unbound omicron SP (Table 3 and Table 4), and therefore suggests that binding generally causes reduced flexibility of residues. This observation is consistent with the results of the average RMSD reported in this study, where binding of the top-ranked compounds and zafirlukast generally enhanced structural stability. Among the bound systems, zafirlukast against omicron SP had the lowest average RMSF value in the whole protein (2.70 Å) and in the RBD (2.25 Å), which might be an indication of its better capability to form stable bonds relative to the investigated compounds and hence its advantage over the test compounds as an inhibitor of the target. This observation contradicts the results of the thermodynamic binding free energy and RMSD findings in this study, where 6-Hydroxycyanidin 3-rutinoside had the best binding free energy and lowest RMSD value. Of the two compounds (geraniin and maysin) with better affinity than zafirlukast, geraniin had the lowest RMSF values in the whole protein (SC-2WT (1.66 Å), omicron (3.26 Å)) and at the RBD (SC-2WT (1.20 Å), omicron (2.62 Å)) (Table 3 and Table 4) of both targets, and hence this suggests its better capability to form stronger bonds with the SP of both targets than maysin. This observation is unlike the binding free energy and RMSD findings of this study where maysin was predicted to have the better affinity for the SP of both strains and the better compound in terms of stability with omicron spike protein. However, the differences in the ROG, RMSD, and RMSF findings observed in this study point to the fact that several parameters may contribute to the total thermodynamic stability of a complex as earlier affirmed by previous studies [28,29,30,33].

**Figure 5:**
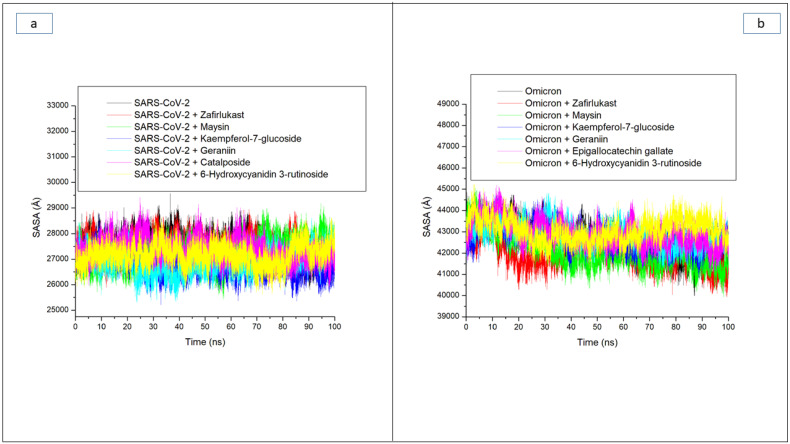
Comparative solvent accessible surface area (SASA) plots of alpha-carbon, top five LOCM compounds, and zafirlukast against the spike protein of (**a**) SARS-CoV-2 wild-type and (**b**) omicron, after 100 ns MD simulation period.

Solvent accessible surface area (SASA) is another important index examining protein folding and changes in a protein’s surface area during simulation, with higher SASA values indicating an increase in surface area and lower SASA values indicating a decrease in protein volume [32]. In this study, minor fluctuations in SASA were observed during the simulation period in the SC-2WT SP systems (Figure 5a). This observation suggests that the binding of the test compounds and zafirlukast to SC-2WT SP had a negligible effect on the surface area and volume of SC-2WT SP. As a result, an overall small variation of 700 Å among all systems was observed, with the Kaempferol-7-glucoside-SC-2WT SP complex (26,835.74 Å) having the lowest value (Table 3). The little impact of binding of the compounds on the SASA values of the resulting complexes follows a similar pattern as the results of ROG values obtained in this study where little impact of the compounds’ binding was noticed on SC-2WT SP folding and, hence, may be an indication that the binding of the investigated compounds and zafirlukast on SC-2WT SP does not disrupt its thermodynamic entropy. While this observation regarding SASA agrees with the findings of Aribisala and Sabiu [23], where binding of the lead phenolics with penicillin-binding protein 2a had negligible effects on SASA values of the resulting complexes, it contradicts the report of Mousavi et al. [32], who reported that the interactions of ligands with proteins can have a significant impact on SASA value. As with the ROG findings against omicron SP, a continuous reduction in SASA value depicting reduced surface area and volume was observed throughout the simulation period for all the systems (Figure 5b). This observation was more prominent with zafirlukast and maysin, with average SASA values of 41,942.55 Å and 41,966.76 Å, respectively, against the protein. The continuous reduction in volume and surface area of omicron SP signifying probable increase in thermodynamic stability is in tandem with the results of RMSD and ROG obtained in this study. Among the two LOCM compounds (maysin and geraniin) with better broad spectrum activity than zafirlukast against the SP of both proteins, maysin (41,966.76 Å) seems to cause better folding and hence better stability of the omicron SP than geraniin (42,988.52 Å), while geraniin, on the other hand, has better stability with SC-2WT SP than maysin (Figure 5a,b). Except for the RMSF findings, this observation is consistent with the other thermodynamic indices explored in this study. It is however noteworthy that, while kaempferol-7-glucoside, with an average SASA value of 26,835.58 Å against SC-2WT SP, is consistent with its binding free energy and RMSD values as the best compound for SC-2WT SP, 6-Hydroxycyanidin 3-rutinoside, with the highest SASA value (43,070.18 Å) against omicron SP, does not correlate with its binding free energy and RMSD as the best compound for the protein. Hence, the increased binding free energy and structural stability observed with 6-Hydroxycyanidin 3-rutinoside relative to zafirlukast and the other investigated compounds might not be due to its ability to induce protein folding as earlier affirmed for the data obtained regarding the ROG values.

**Figure 6:**
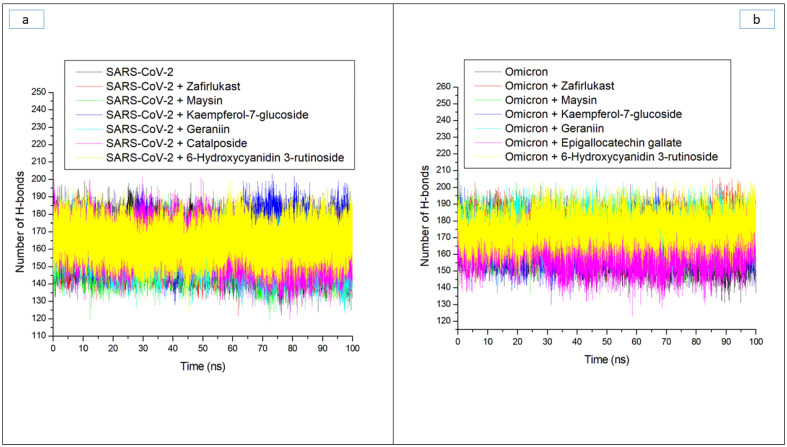
Time evolution of the number of hydrogen bonds in (**a**) SARS-CoV-2, and (**b**) Omicron spike proteins following binding of top five LOCM compounds and zafirlukast during the 100 ns MD simulation period.

**Figure 7:**
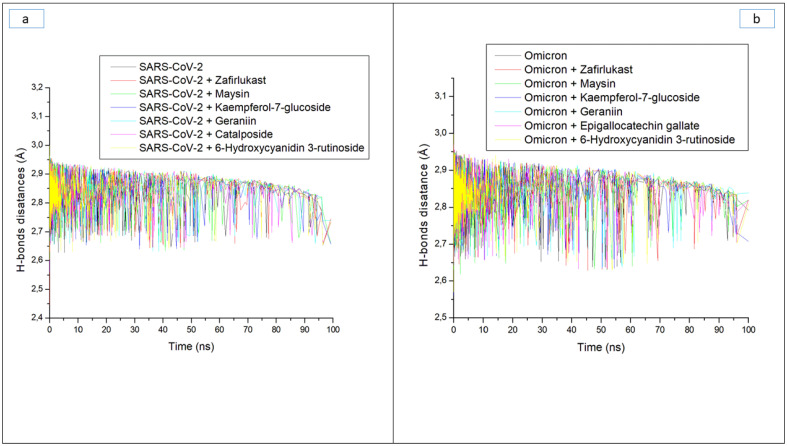
Time evolution of the hydrogen bond distance in (**a**) SARS-CoV-2, and (**b**) Omicron spike proteins following binding of top five LOCM compounds and zafirlukast during the 100 ns MD simulation period.

**Figure 8:**
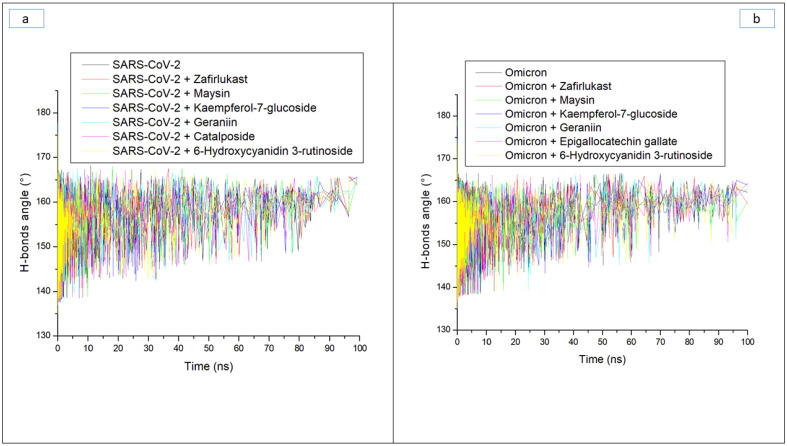
Time evolution of the hydrogen bond angles in (**a**) SARS-CoV-2, and (**b**) Omicron spike proteins following binding of top five LOCM compounds and zafirlukast during the 100 ns MD simulation period.

Hydrogen bonds and their respective distance are important for the stability of a protein structure and can therefore be evaluated during simulation to understand the influence of ligand binding on the stability of a protein [28,33]. Figure 6a and b display a stable fluctuation in the pattern of the number of hydrogen bonds formed in SC-2WT and omicron SP, respectively, during the simulation. Generally, except for epigallocatechin gallate-omicron complex, binding results in an increased number of hydrogen bonds in SC-2WT and omicron SP (Table 3). This observation suggests the occupancy of some intramolecular space of the proteins by the ligands, and the increased hydrogen bonds could mean hydrogen bond interactions contributed as a result of intermolecular bindings. Interestingly, 6-Hydroxycyanidin 3-rutinoside and kaempferol-7-glucoside, with the best binding free energy against omicron and SC-2WT SP, respectively, also had the highest number of hydrogen bonds against the respective target (Table 3). This observation highlights the impact of hydrogen bond interactions in the observed binding free energy of the best compound identified with the SP of both targets and correlates the previous findings of Aribisala et al. [23], where the best phenolics instigated the highest number of total hydrogen bonds in penicillin-binding protein 2a following 120 ns MD simulation. Similarly, following the binding of the two compounds (geraniin and maysin) with better affinity than zafirlukast, the complexes in each case of the SP had a higher number of hydrogen bonds than zafirlukast complexes against both targets. This observation further highlights the impact of hydrogen bond interactions in the observed higher broad-spectrum of activities of geraniin and maysin compared to zafirlukast. Between the two compounds, omicron SP-maysin complex (174.83) had higher hydrogen bond interactions than omicron SP-geraniin complex (172.72) and vice versa against SC-2WT. This finding pinpoints the influence of hydrogen bonds in the observed better affinity of maysin for omicron SP than SC-2WT and vice versa for geraniin. Figure 7a,b displays a continued reduction in hydrogen bond distance in SC-2WT and omicron SP, respectively, as the simulation progresses that is similar in all the systems (unbound SP and top-ranked LOCM-SP complexed systems). All the systems had an average hydrogen bond distance of 2.85 Å and 2.87 Å, respectively, against SC-2WT and omicron SP, which suggests that the binding of both targets does not disrupt the arrangement and geometry of both SPs, but rather causes more internal pull between atoms and residues of the SPs as the simulation progress. This observation further corroborates the thermodynamic compatibility of the top-ranked LOCM compounds and zafirlukast with the targets observed in this study as established by the RMSD findings. Figure 8a and b display a stable fluctuation of hydrogen bond angle in SC-2WT and omicron SP, respectively, as the simulation progresses. At 0 ns, a wide range of swaying between 138° and 167° was observed in SC-2WT and omicron SP plots that was lesser as the simulation progressed. At 100 ns, the hydrogen bond angle fluctuation was between 157° and 167° for SC-2WT SP plot systems and 154° and 167° for omicron SP systems. The ideal hydrogen bond angle is 180° [34]; thus, the lesser fluctuation of the hydrogen bond angle towards 180° as the simulation progresses indicates the better strength of the hydrogen bonds, which correlates with the hydrogen bond distance findings of this study. Against omicron SP, all the systems had average hydrogen bond angle values of between 151.66° and 152.02° with a negligible variance of 0.36°, which suggests little impact of the parameter in the discriminations of the binding free energy observed in this study. However, against SC-2WT, an average swaying of between 157.95° and 165.21° was observed among all the systems, with kaempferol-7-glucoside having the closer value to 180°. This observation revealed the better strength of the hydrogen bonding in SC-2WT SP compared to omicron SP following binding of the top-ranked compounds and zafirlukast that was stronger in the kaempferol-7-glucoside-SC-2WT SP complex. The stronger hydrogen bonding in the kaempferol-7-glucoside-SC-2WT SP complex could have contributed to the observed higher binding free energy observed in the complex relative to other complexes.

In silico assessment of a compound’s ADMET properties gives insight into the compound’s in vivo pharmacological friendliness, bioavailability, and relative toxicity when employed as an oral medication, and this has been found to help minimize the risk of disapproval during the pre- and clinical phases of drug development [35]. Lipinski’s rule of five restricts the molecular weight, hydrogen donor, hydrogen acceptor, and octanol co-efficient of a bioactive drug to 500 g/mol, 5, 10, and 5, respectively [36]. This rule is used to assess the drug-likeness of compounds with pharmacological activity. In this study, the investigated compounds and the zafirlukast had numbers of hydrogen bonds and acceptors that were above the required limit of the rule of five. Also, except for epigallocatechin gallate (458.3 g/mol) and catalposide (482.4 g/mol), the investigated compounds and zafirlukast all had molecular weights that were higher than 500 g/mol (Table 5). Lipinski et al. [36] predicted that compounds with less than two violations of the rule of five would have good oral drug-likeness properties with pharmacological activity. Thus, the observation that all the investigated LOCM compounds and zafirlukast failed Lipinski’s rule calls for structural alterations/modifications aimed at reduction in molecular weight, the number of hydrogen acceptors, and donors to a level that will allow for enhanced pharmacological activity. Nevertheless, all the investigated compounds and zafirlukast are soluble in water and can be easily transported through systemic circulation [37]. Similarly, their low bioavailability scores of 0.17 suggest that they may be used as oral drugs [14]. Cytochrome P450 (CYP), being an important isoenzyme in drug metabolism, plays a key role in drug toxicity [38]. Remarkably, all of the investigated LOCM compounds and zafirlukast do not inhibit any of the CYP isoenzymes (CYP1A2, CYP2C19, CYP2C9, CYP2D6, and CYP2A4) (Table 5) that are important in phase I drug detoxification in the liver and this suggests that they may not cause drug–drug interaction when co-administered with other drugs in the liver. Except for maysin, geraniin, and 6-Hydroxycyanidin 3-rutinoside, which were predicted to be immunotoxic, the others were predicted to be non-toxic for all the toxicity endpoints investigated. Zafirlukast, on the other hand, was predicted to be immunotoxic and hepatotoxic. With the exception of geraniin, which belongs to toxicity class three with a high LD_50_ value of 300 mg/kg that is similar to zafirlukast, all the other investigated compounds belong to the toxicity class of drugs that can be deployed as medications with lower LD_50_ values (1000–5000 mg/kg) than zafirlukast (Table 5).

**Figure 9:**
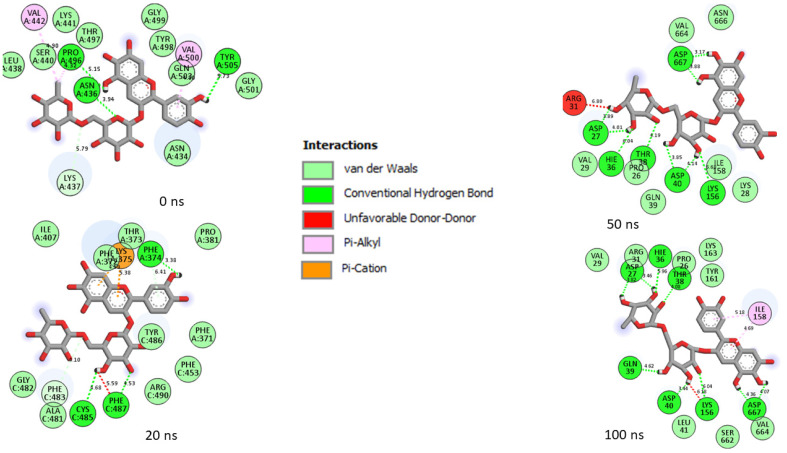
Plot of interactions of 6-Hydroxycyanidin 3-rutinoside against the SP of omicron at different timeframes during the 100 ns simulation. At 0 ns, residues such as 496, 505, 501, 500, 498, and 493 reported in the RBD of omicron were observed in the interaction. As the simulation progressed, changes in the amino acid interactions were observed as seen in the 20 ns, 50 ns, and 100 ns interaction frames. However, common amino acids such as Asp40, Asp667, Asp27, Hie36, Ile158, Pro26, Thr38, and Val29 can be observed in the interactions at timeframes 50 and 100 ns.

**Figure 10:**
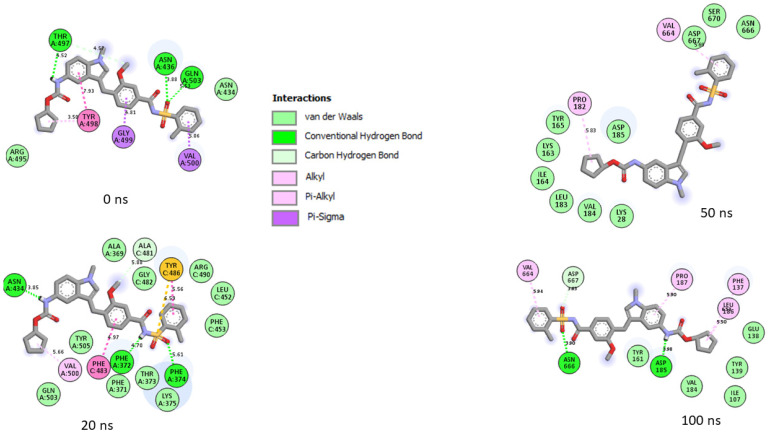
Plot of interactions of zafirlukast against the SP of omicron at different timeframes during the 100 ns simulation. At 0 and 20 ns, RBD residues such as 505, 500, 498, and 503 were observed in the interaction plot. As the simulation progressed, a significant change in the amino acid interactions was observed as seen in the 50 ns and 100 ns interaction frames. However, common amino acids such as Val184, Asp185, and Asn666 can be observed in the interactions at timeframes 50 and 100 ns. Noteworthy also is the significant reduction in the number of interactions after 20 ns, which could have contributed to the reduced binding free energy observed in the complex relative to 6-Hydroxycyanidin 3-rutinoside against the target.

**Figure 11:**
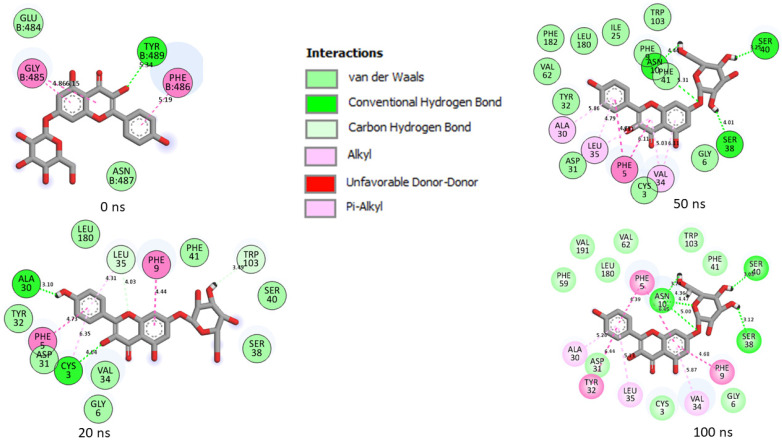
Plot of interactions of Kaempferol-7-glucoside against the SP of SC-2WT at different timeframes during the 100 ns simulation. At 0 ns, RBD residues such as 489, 486, 484 and 487 could be seen in the interaction plot of the complex. At 20 ns, a significant change in the amino acid interactions took place that was consistent throughout the simulation and, as a result several common amino acid residues, could be observed at the interaction timeframes of 20 ns, 50 ns and 100 ns.

**Figure 12:**
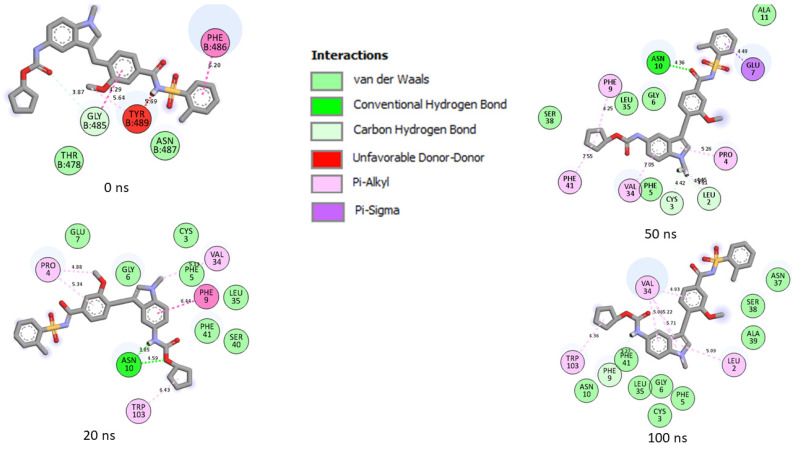
Plot of interactions of zafirlukast against the SP of SC-2WT at different timeframes during the 100 ns simulation. At 0 ns, RBD residues such as 489, 486, and 487 could be seen in the interaction plot of the complex. At 20 ns, a significant change in the amino acid interactions occurred that was consistent throughout the simulation and, as a result, several common amino acids could be observed at the interaction timeframes of 20 ns, 50 ns and 100 ns.

Generally, a ligand’s capacity to bind and inactivate a protein is governed by many thermodynamic parameters relating to amino acid residue flexibility, protein stability, compactness, and, most critically, the nature of contacts with essential amino acids [35,39,40]. Thus, in this study, the number, nature, and length of bond interactions formed by the top five LOCM compounds and zafirlukast with SC-2WT and omicron SP after 100 ns were analyzed. The plot of interactions at different timeframes of interactions for zafirlukast and LOCM compounds with the highest binding free energy against SPs of omicron and SC-2WT are presented in Figure 9, Figure 10, Figure 11 and Figure 12, while the plots of interactions after 100 ns for the other top LOCM compounds are presented in Table S2. The nature, bond lengths, and numbers of interactions of the top five LOCM compounds and zafirlukast against the two investigated SPs vary from one compound to another and were noted to have impacts on the binding free energy observed in this study. Bond interactions including hydrogen bonds (conventional and carbon), Van der Waals, amide π-stacked, π-anion, π-sigma, π-cation, π-π t-cation, π-π t-shaped, π-π t-stacked, π-alkyl, alky, π-Sulphur, and some unfavorable donor–donor interactions were observed. Specifically, maysin interacting with the highest number of interactions (26) with omicron SP does not justify its lower binding free energy relative to 6-Hydroxycyanidin 3-rutinoside (19 interactions (Figure 9)) which has the highest binding free energy. However, when the nature of the interactions between both compounds with omicron SP was further analyzed, the presence of two unfavorable donor–donor interactions in the maysin–omicron SP complex interactions, indicative of repulsive forces [24,40], might have drastically reduced the binding free energy score of maysin against omicron SP relative to 6-Hydroxycyanidin 3-rutinoside with one unfavourable bond (Figure 9, Table 6). Furthermore, the maysin–omicron SP complex (4.89 Å) had a higher average bond length than 6-Hydroxycyanidin 3-rutinoside (4.65 Å) against omicron SP. Longer bond lengths signify less pull between two intra- or inter-molecules [28], and the longer bond lengths between maysin and omicron SPs relative to 6-Hydroxycyanidin 3-rutinoside against omicron SP could have further drastically reduced the effects of the higher number of interactions between maysin and omicron SPs observed in this study. Despite the diminishing factors in the maysin and omicron SP interactions, maysin still had better binding free energy than the other LOCM compounds and zafirlukast (Figure 10), indicating how promising the compound is as an omicron SP inhibitor. Interestingly, except for kaempferol-7-glucoside (two interactions with one hydrogen contact) against omicron SP, all the investigated top-ranked compounds had higher numbers of interactions and hydrogen bond contacts than zafirlukast (12 interactions with three hydrogen bond contacts) (Table 6). This observation agrees with the binding free energy findings in this study, where kaempferol-7-glucoside was the only compound with a lower binding free score than zafirlukast and, hence, emphasizes the importance of the number of interactions and hydrogen bond contacts in binding free energy determination as earlier reported [31]. Furthermore, all the investigated compounds had lower average bond length interactions against omicron SP relative to zafirlukast + omicron SP (Figure 10, Table 6), further highlighting the importance of bond length in the determination of binding free energy scores. Unlike the omicron SP plots with the investigated compounds, Kaempferol-7-glucoside with the highest binding free energy against SC-2WT SP also had the highest number of interactions (21), seven of which are important interactions with Tyr32, Phe9, Val34, Leu35, Phe5, Ala30, and Val34 (Figure 11, Table 6). Furthermore, with the exception of 6-Hydroxycyanidin 3-rutinoside + SC-2WT SP (4.60 Å), Kaempferol-7-glucoside (4.62 Å) interacted with the lowest average bond lengths against SC-2WT SP. These observations further lend credence to the importance of the number, nature, and length of interaction in the higher binding free energy score observed in the Kaempferol-7-glucoside interaction plot with SC-2WT SP. However, zafirlukast having a lower number of interactions and hydrogen bond contacts against SC-2WT SP (Figure 12, Table 6) relative to catalposide and 6-Hydroxycyanidin 3-rutinoside does not justify its higher binding free energy than its complexes with SC-2WT SP (Table 6). However, the higher number of important interactions (6) with Val34 (4), Trp103, and Leu2 observed in the zafirlukast + SC-2WT SP plot (Figure 12) relative to five and three interactions formed between 6-Hydroxycyanidin 3-rutinoside and catalposide, respectively, against SC-2WT SP (Table S2 and Table 6) could have partially contributed to the higher binding free energy of zafirlukast against SC-2WT SP. Bond analysis into the interaction plots of maysin and geraniin (the two LOCM compounds with higher binding free energy than zafirlukast against SP of omicron and SC-2WT) revealed why they had better broad spectrum activities than zafirlukast and why maysin was more thermodynamically compatible with omicron SP and vice versa for geraniin against SC-2WT. Against omicron, both compounds had higher numbers of interactions (maysin (26), geraniin (15)) and hydrogen contacts (maysin (nine), geraniin (four)) with lower average bond lengths of interaction (maysin (4.89 Å), geraniin (4.69 Å)) than zafirlukast with maysin having the highest number of interactions and hydrogen bond contacts (Table 6). Similarly, against SC-2WT, maysin, and geraniin had higher numbers of hydrogen bond contacts (maysin (4), geraniin (8)) with significantly lower average bond length of interaction (maysin (4.72 Å), geraniin (4.67 Å)) than zafirlukast (Table 6) with geraniin having the highest in both cases. Hence, the better broad spectrum of activities observed with maysin and geraniin relative to zafirlukast against SP of omicron and SC-2WT could be attributed to the number, type, and bond length of interactions with the SP of both variants. Similarly, the same features in the plots of both compounds with omicron and SC-2WT SP could also account for why maysin was more thermodynamically compatible with omicron SP and vice versa for geraniin against SC-2WT. Due to the dynamic nature of MD simulation, where both components of the complex are flexible [41], a significant periodical shift was depicted by the investigated compounds during the simulation period. This observation is consistent with the report of Al-karmalawy et al. [42], where alacepril had significant shift toward a transient opened cleft on hACE2 during a 70 ns simulation period. Consequently, in this study, the amino acid residues in the interaction plots for zafirlukast complexes, as well as the investigated top five compounds, differ periodically during the 100 ns simulation period (as seen in the plot of interactions of the SPs with zafirlukast and LOCM compounds with the highest binding free energy (Figure 9, Figure 10, Figure 11 and Figure 12)), and from those observed from the molecular docking studies at the RBD of both SPs. However, after 100 ns, Asp40, which forms hydrogen bonds with 6-Hydroxycyanidin 3-rutinoside, geraniin, and maysin, and ASP667, which forms hydrogen bonds with 6-Hydroxycyanidin 3-rutinoside and epigallocatechin gallate, are important amino acids in the interactions observed in this study. Similarly, except for zafirlukast, Asn10 of SC-2WT, which forms hydrogen bond interactions with all of the investigated LOCM compounds, is important in the interactions with the top-ranked LOCM compounds.

**Figure 13:**
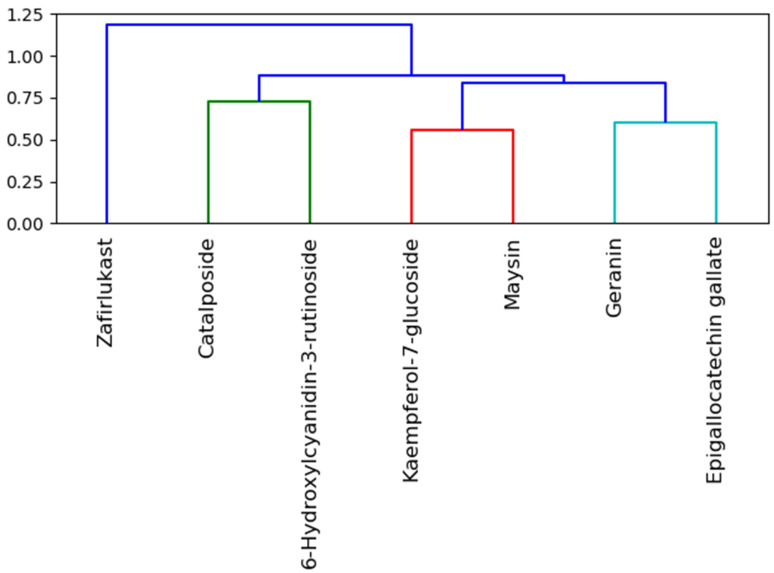
Similarity clustering of the top-ranked LOCM compounds and zafirlukast. Clusters in the dendrogram are color-coded with molecules with the same color and clusters more similar than those with different colors and clusters. Also, the horizontal axis displays the similarity score, and the lesser the similarity scores of two compounds, the more similar they are to one another.

Analysis of similarity clustering of the investigated compounds revealed that zafirlukast, with the highest similarity score (1.20), belongs to a different cluster (with different colors) relative to the top five LOCM compounds and hence suggests that zafirlukast (Figure 13) is structurally different from all of the top-ranked LOCM compounds [43]. This observation is not surprising, as zafirlukast is a synthetic peptide leukotriene receptor inhibitor while LOCM compounds are natural compounds from plants. Even though all the investigated compounds and zafirlukast failed the Lipinski rule of five due to the high molecular weights, numbers of hydrogen bond acceptors, and donors, the difference in structure between zafirlukast and the LOCM compounds could have impacted the higher toxicity observed with zafirlukast. Zafirlukast was the only compound that was hepatotoxic and except for geraniin, zafirlukast belongs to a toxicity class three with a high LD_50_ of 300 mg/kg. Toxicity category three is slightly toxic and irritating and could result in adverse side effects in the host when used as a drug [35,44]. This observation, however, suggests the advantages of the top-ranked LOCM compounds as less toxic drugs. All of the top-ranked LOCM compounds had a smaller range of similarity scores between 0.6 and 0.75 while belonging to different clusters and colors (Figure 13). The smaller range of similarity scores between the investigated LOCM compounds suggests to a certain degree that they had some common moieties and a closer look into their structural compositions revealed they are all phenolic compounds with either paragallol, resorcinol, or catechol structures. 6-Hydroxycyanidin 3-rutinoside and cataposide, kaempferol-7-glucoside and maysin, as well as geraniin and epigallocatechin gallate, were all clustered differently with different colors highlighting the slight difference in their structure despite close similarity scores. This observation, however, had little impact on the affinity for omicron and SC-2WT SP and the pharmacokinetic properties observed with the LOCM compounds.

## 4. Conclusions

This study identified lead compounds from LOCM with the ability to inhibit omicron and SC-2WT SPs. In particular, 6-hydroxycyanidin-3-rutinoside and kaempferol-7-glucoside had the best affinity towards the SP of omicron and SC-2WT, respectively. Of the 73 LOCM compounds examined, only maysin and geraniin had better broad-spectrum affinities for the SP of omicron and SC-2WT than zafirlukast. This observation suggests the advantages of maysin and geraniin over zafirlukast and the other LOCM compounds in treating COVID-19 infections that may arise as a result of either strain. It was also observed that the high-ranked LOCM compounds were more thermodynamically compatible with SC-2WT than with omicron SP, with 6-hydroxycyanidin3-rutinoside and kaempferol-7-glucoside being the most thermodynamically stable with omicron and SC-2WT SPs, respectively. This observation further substantiates the promising affinity of 6-hydroxycyanidin3-rutinoside and kaempferol-7-glucoside for omicron and SC-2WT SPs, respectively. The thermodynamic compatibility with the SP of both strains was attributed to the number, type, and length of the bonds formed between the compounds and the SPs. Except for geraniin, the top-ranked compounds were predicted with a low toxicity profile compared to zafirlukast, and this was attributed to their phenolic moieties. However, structural modification of the LOCM compounds and zafirlukast is required to drastically improve their drug-like and pharmacological properties. Also, the in vitro and in vivo confirmation of the observed activities with the identified compounds in this study is highly recommended, and efforts taken in this direction would undoubtedly help identify potential anti-COVID-19 drug candidates.

## Figures and Tables

**Table 1:** Docking scores of top five LOCM compounds and standards against omicron and SC-2WT SP variants.

Ligands	Binding Affinity (kcal/mol)
Omicron variant SP	
Zafirlukast	−7.4
Cefoperazone	−6.2
Maysin	−7.5
6-Hydroxylcyanidin-3-rutinoside	−7.2
Kaempferol-7-glucoside	−7.2
Geraniin	−7.5
Epigallocatechin gallate	−7.2
SC-2WT SP	
Zafirlukast	−7.9
Cefoperazone	−6.7
Maysin	−8.4
Geraniin	−7.1
Catalposide	−7.4
Kaempferol-7-glucoside	−7.3
6-Hydroxylcyanidin-3	−7.2

**Table 2:** Energy components (kcal/mol) of the top-ranked LOCM compounds against SP of omicron and SC-2WT.

Energy Components (kcal/mol)					
Complex	ΔE_vdW_	ΔE_elec_	ΔG_gas_	ΔG_solv_	ΔG_bind_
Omicron SP					
6-Hydroxycyanidin 3-rutinoside	−26.63 ± 9.10	−137.98 ± 33.21	−164.62 ± 29.55	121.65 ± 23.84	−42.97 ± 8.34
Epigallocatechin gallate	−22.16 ± 6.10	−58.77 ± 18.02	−80.93 ± 15.53	53.06 ± 11.68	−27.86 ± 5.98
Geraniin	−34.44 ± 4.42	−31.07 ± 10.71	−65.52 ± 11.96	34.23 ± 8.83	−31.28 ± 7.25
Kaempferol-7-glucoside	−21.98 ± 5.45	−36.20 ± 20.45	−58.19 ± 21.36	38.87 ± 17.02	−19.31 ± 5.33
Maysin	−43.96 ± 6.71	−59.86 ± 17.91	−103.83 ± 20.81	64.94 ± 12.05	−38.88 ± 10.21
Zafirlukast	−36.22 ± 6.65	−23.79 ± 13.96	−60.02 ± 15.90	37.64 ± 13.87	−22.38 ± 5.95
SC-2WT SP					
6-Hydroxycyanidin 3-rutinoside	−37.69 ± 5.97	26.06 ± 28.31	−11.63 ± 29.43	−17.20 ± 19.81	−28.84 ± 10.87
Catalposide	−37.96 ± 3.84	−22.18 ± 9.58	−60.14 ± 11.44	31.23 ± 7.65	−28.90 ± 4.95
Geraniin	−36.41 ± 4.51	−45.64 ± 11.29	−82.07 ± 11.43	45.16 ± 8.53	−36.90 ± 4.55
Kaempferol-7-glucoside	−47.06 ± 6.40	−20.68 ± 8.10	−67.75 ± 10.97	30.64 ± 5.15	−37.11 ± 7.01
Maysin	−35.97 ± 7.15	−38.54 ± 10.88	−74.51 ± 10.98	41.66 ± 6.49	−34.85 ± 6.01
Zafirlukast	−44.23 ± 5.55	−14.70 ± 8.92	−58.94 ± 11.24	25.21 ± 7.82	−33.73 ± 4.99

ΔE_vdW_ = van der Waals energy; ΔG_bind_ = total binding free energy; ΔE_gas_ = gas phase free energy; ΔE_elec_ = electrostatic energy; ΔG_sol_ = solvation free energy.

**Table 3:** Mean RMSD, ROG, RMSF, and SASA of apo-spike proteins, top five LOCM compounds, and standard against SP of omicron and SC-2WT.

Systems	Average RMSD (Å)	Average RMSF (Å)	Average ROG (Å)	Average SASA (Å)	Average Number of H-Bonds	Average Distance (Å) of H-Bonds	Average Angle (°) of H-Bonds
Omicron SP							
6-Hydroxycyanidin 3-rutinoside	5.94 ± 1.32	3.91 ± 1.75	39.50 ± 0.42	43,070.18 ± 533	176.16 ± 8.4	2.87 ± 0.04	151.73 ± 7.8
Epigallocatechin gallate	7.60 ± 1.61	3.28 ± 1.78	39.12 ± 0.67	42,933.95 ± 612	162.78 ± 8.4	2.87 ± 0.04	151.66 ± 7.4
Geraniin	8.71 ± 1.70	3.26 ± 1.48	38.58 ± 0.44	42,988.52 ± 552	172.74 ± 8.3	2.87 ± 0.04	151.89 ± 7.7
Kaempferol-7-glucoside	9.44 ± 2.50	3.91 ± 1.82	38.12 ± 0.71	42,578.88 ± 587	168.86 ± 8.3	2.87 ± 0.04	152.02 ± 7.6
Maysin	8.25 ± 2.14	3.91 ± 2.41	38.20 ± 0.68	41,966.76 ± 761	174.83 ± 8.4	2.87 ± 0.04	151.84 ± 7.3
Zafirlukast	6.99 ± 1.00	2.70 ± 1.30	38.41 ± 0.45	41,942.55 ± 578	171.01 ± 8.6	2.87 ± 0.04	151.66 ± 7.4
Apo omicron	8.56 ± 2.14	4.43 ± 1.82	38.07 ± 0.69	42,060.34 ± 865	163.40 ± 7.8	2.87 ± 0.04	151.93 ± 7.6
SC-2WT SP							
6-Hydroxycyanidin 3-rutinoside	2.43 ± 0.45	1.66 ± 0.85	30.48 ± 0.28	27,171.49 ± 396	162.74 ± 8.3	2.85 ± 0.06	164.74 ± 8.4
Catalposide	2.67 ± 0.36	1.77 ± 1.02	30.18 ± 0.29	27,394.87 ± 439	161.02 ± 9.3	2.85 ± 0.06	161.02 ± 9.33
Geraniin	2.75 ± 0.44	1.66 ± 0.89	30.09 ± 0.29	26,914.74 ± 433	165.32 ± 7.3	2.85 ± 0.05	158.31 ± 8.7
Kaempferol-7-glucoside	2.64 ± 0.43	1.78 ± 0.81	30.28 ± 0.33	26,835.58 ± 418	166.81 ± 8.7	2.85 ± 0.06	165.21 ± 8.7
Maysin	3.07 ± 0.49	1.92 ± 1.14	30.39 ± 0.33	27,277.10 ± 491	163.32 ± 6.4	2.85 ± 0.06	157.95 ± 8.3
Zafirlukast	3.15 ± 0.58	1.70 ± 0.76	30.16 ± 0.26	27,473.62 ± 390	161.21 ± 7.3	2.85 ± 0.05	160.26 ± 8.2
Apo SC-2WT	2.48 ± 0.33	1.64 ± 0.83	30.27 ± 0.34	27,541.98 ± 449	159.24 ± 8.3	2.85 ± 0.06	161.56 ± 8.7

**Table 4:** Average RMSF (Å) of omicron and SC-2WT SP RBD residues following binding with top-ranked LOCM compounds and Zafirlukast.

RBD Residues of Omicron SP	6-Hydroxycyanidin 3-Rutinoside	Epigallocatechin Gallate	Geraniin	Kaempferol-7-Glucoside	Maysin	Zafirlukast	Apo-Omicron SP
353	3.64	2.10	2.94	2.45	2.17	2.12	3.17
493	2.56	1.51	1.89	2.53	2.23	1.60	3.17
496	2.83	2.13	2.36	2.25	2.53	1.95	3.47
498	2.50	1.95	1.88	1.77	2.13	2.27	2.89
500	2.64	2.22	2.31	2.01	2.42	2.61	3.15
501	3.00	2.76	2.85	2.16	2.78	2.78	3.60
505	2.89	3.76	4.16	3.02	3.21	2.45	3.88
Total RMSF	2.86	2.34	2.62	2.31	2.49	2.25	3.33
**RBD Residues of SC-2WT SP**	**6-Hydroxycyanidin 3-Rutinoside**	**Catalposide**	**Geraniin**	**Kaempferol-7-Glucoside**	**Maysin**	**Zafirlukast**	**Apo-SC-2WT SP**
473	1.12	1.22	1.03	1.34	1.18	1.14	1.08
475	1.45	1.44	1.35	1.59	1.58	1.41	1.26
478	1.44	1.36	1.25	1.63	1.40	1.51	1.07
484	1.32	1.24	1.21	1.37	1.36	1.39	1.14
486	1.29	1.19	1.17	1.34	1.36	1.36	1.08
487	1.35	1.11	1.24	1.27	1.43	1.36	1.21
489	1.23	1.25	1.16	1.42	1.42	1.34	1.08
Total RMSF	1.31	1.26	1.20	1.42	1.39	1.35	1.13

**Table 5:** ADMET properties of all the top five LOCM compounds investigated against SP of each of the target.

Ligands	MW < 500 (g/mol)	HB- A ≤ 10	HB- D ≤ 5	Log *P* o/w ≤ 5	WS	GI Absorption	BBB Permeant	Pgp	Inhibitor of CYP 450 s					LV (N)	BS	H	C	IM	M	CY	LD_50_ (mg/kg)	TC
									CYP 1A2	CYP 2C19	CYP 2C9	CYP 2D6	CYP 3A4									
6-Hydroxycyanidin 3-rutinoside	611.53	16	11	−2.45	S	L	N	N	N	N	N	N	N	Y (3)	0.17	I	I	A	I	I	5000	5
Epigallocatechin gallate	458.3	11	8	1.01	S	L	N	N	N	N	N	N	N	Y (2)	0.17	I	I	I	I	I	1000	4
Catalposide	482.4	12	6	−0.90	S	L	N	Y	N	N	N	N	N	Y (2)	0.17	I	I	I	I	I	2000	4
Geraniin	952.6	27	14	−1.70	S	L	N	Y	N	N	N	N	N	Y (2)	0.17	I	I	A	I	I	300	3
Kaempferol-7-glucoside	952.6	27	14	−1.70	S	L	N	Y	N	N	N	N	N	Y (2)	0.17	I	I	I	I	I	5000	5
Maysin	952.6	27	14	−1.70	S	L	N	Y	N	N	N	N	N	Y (3)	0.17	I	I	A	I	I	5000	5
Zafirlukast	448.3	11	7	−0.04	S	L	N	N	N	N	N	N	N	Y (2)	0.17	A	I	A	I	I	300	3

Keys: MW: Molecular weight; BBB permeant: Blood-brain barrier permeation; HB-D: Hydrogen bond donor; Log *P* o/w: Partition coefficient; HB-A: Hydrogen bond acceptor; CY: Cytotoxicity; Pgp substrate: Permeability glycoprotein substrate; WS: Water solubility; L: Low; H: High; CYP: Cytochrome; LV: Lipinski violation; BS: Bioavailability score; H: Hepatotoxicity; C: Carcinogenicity; IM: Immunotoxicity; GI absorption: Gastrointestinal absorption; M: Mutagenicity; LD: Lethal dose; TC: Toxicity class; MS: Moderate soluble; S: Soluble; VS: Very soluble; PS: poorly soluble; IN: Insoluble; N: No; Y: Yes; A: Active; I: Inactive, N: number of violation.

**Table 6:** Molecular interactions between the top five LOCM compounds against SP of omicron and SC-2WT after 100 ns of simulation.

Top Five LOCM Compounds	Total Number of Interactions (Average Distance)	Number of Hydrogen Bonds (Average Distance) and Interaction Residues	Other Important Interactions and Residues	Unfavorable Bonds
Omicron SP				
6-Hydroxycyanidin 3-rutinoside	19 (4.65 Å)	9 (4.42 Å) [Gln39, Asp40, Lys156, Asp667 (2), Tyr38, Hie36, Asp27 (2)]	2 (4.94 Å) [Ile158 (2)]	None
Epigallocatechin gallate	14 (4.29 Å)	7 (4.16 Å) [Tyr161, Asp667 (2), Ser662 (2), Arg671, Asp185]	2 (4.76 Å) [Tyr161(2)]	None
Geraniin	15 (4.69 Å)	4 (4.33 Å) [Asn157, Asp40, Pro220, Lys652]	4 (5.05 Å) [Asp40, Leu41, Ile158, Pro220]	None
Kaempferol-7-glucoside	2 (4.52 Å)	1 (4.52) [Lys28]	None	None
Maysin	26 (4.89 Å)	9 (4.26 Å) [Asp461, Ser655 (2), Val651 (2), Asp40 (2), Lys156 (2)	7 (5.55 Å) [Asp461, Asp185, Ile158(3), Asp40, Lys156]	2 [Leu41, Lys156]
Zafirlukast	12 (5.05 Å)	3 (3.83 Å) [Asn666, Asp667, Asp185]	4 (5.96 Å) [Val664, Pro187, Phe137, Leu186]	None
SC-2WT SP				
6-Hydroxycyanidin 3-rutinoside	18 (4.60 Å)	6 (4.08 Å) [Asp31 (3), Ala39, Asn10, Ser40]	5 (5.45 Å) [Leu180, Tyr32, Phe9, Val32 (2)]	None
Catalposide	15 (5.07 Å)	4 (4.33 Å) [Asn10, Gly6, Asp31 (2)]	3 (6.28 Å) [Val34, Phe9, Trp103]	None
Geraniin	17 (4.67 Å)	8 (4.21 Å) [Tyr12, Arg13, Ser66, Ala64, Asn21 (2), Glu7, Asn10]	5 (5.48 Å) [Ala11 (3), Lys23, Val8]	1 [Lys23]
Kaempferol-7-glucoside	21 (4.62 Å)	5 (3.88 Å) [Asn10 (3), Ser40, Ser38]	7 (5.46 Å) [Tyr32, Phe9, Val34, Leu35, Phe5, Ala30, Val34]	None
Maysin	13 (4.72 Å)	4 (3.49 Å) [Asn10, Glu7 (2), Gly6]	4 (5.96 Å) [Trp103, Phe41, Phe9, Pro4]	None
Zafirlukast	16 (4.80 Å)	1 (3.27 Å) [Phe9]	6 (5.22 Å) [Val34 (4), Trp103, Leu2]	

## Data Availability

The data are contained within the article or supplementary material.

## Supplementary Materials

The following supporting information can be downloaded at: https://www.mdpi.com/article/10.3390/metabo12100982/s1. Table S1: Docking scores of 73 LOCM compounds against spike protein of SC-2WT and its omicron variant; Table S2: 2D interaction plots of top five LOCM compounds against SP of omicron and SC-2WT after 100 ns simulation

## References

[1] Mokhtari T., Hassani F., Ghaffari N., Ebrahimi B., Yarahmadi A., Hassanzadeh G. (2020). COVID-19 and multiorgan failure: A narrative review on potential mechanisms. J. Mol. Histol..

[2] Renu K., Prasanna P.L., Valsala G.A. (2020). Coronaviruses pathogenesis, comorbidities and multi-organ damage—A review. Life Sci..

[3] Islam T., Hasan M., Rahman M.S., Islam M.R. (2022). Comparative evaluation of authorized drugs for treating Covid-19 patients. Health Sci. Rep..

[4] Şenay Ş. (2020). Coronavirus pandemic and cardiovascular issues. Turk. Gogus Kalp Damar Cerrahisi Derg..

[5] V’kovski P., Kratzel A., Steiner S., Stalder H., Thiel V. (2021). Coronavirus biology and replication: Implications for SARS-CoV-2. Nat. Rev. Microbiol..

[6] Gordon D.E., Jang G.M., Bouhaddou M., Xu J., Obernier K., White K.M., O’Meara M.J., Rezelj V.V., Guo J.Z., Swaney D.L. (2020). A SARS-CoV-2 protein interaction map reveals targets for drug repurposing. Nature.

[7] Vardhan S., Sahoo S.K. (2022). Computational studies on the interaction of SARS-CoV-2 Omicron SGp RBD with human receptor ACE2, limonin and glycyrrhizic acid. Comput. Biol. Med..

[8] Gu H., Krishnan P., Ng D.Y.M., Chang L.D.J., Liu G.Y.Z., Cheng S.S.M., Hui M.M.Y., Fan M.C.Y., Wan J.H.L., Lau L.H.K. (2022). Probable transmission of SARS-CoV-2 omicron variant in quarantine hotel, Hong Kong, China, November 2021. Emerg. Infect. Dis..

[9] Ahmad S., Zahiruddin S., Parveen B., Basist P., Parveen A., Gautam G., Parveen R., Ahmad M. (2021). Indian medicinal plants and formulations and their potential against COVID-19-preclinical and clinical research. Front Pharm..

[10] Rasool N., Bakht A., Hussain W. (2021). Analysis of inhibitor binding combined with reactivity studies to discover the potentially inhibiting phytochemicals targeting chikungunya viral replication. Curr. Drug Discov. Technol..

[11] Sabiu S., O’Neill F.H., Ashafa A.O.T. (2017). Toxicopathological evaluation of a 28-day repeated dose administration of *Zea mays* L. (Poaceae), Stigma maydis aqueous extract on key metabolic markers of Wistar rats. Trans. R. Soc. S. Afr..

[12] Volpato G., God´ınez D., Beyra A., Barreto A. (2009). Uses of medicinal plants by Haitian immigrants and their descendants in the Province of Camag¨uey, Cuba. J. Ethnobiol. Ethnomed..

[13] Balogun F.O., Sabiu S. (2021). A Review of the Phytochemistry, Ethnobotany, Toxicology, and Pharmacological Potentials of Crescentia cujete L. (Bignoniaceae). Evid. Based Complement. Altern. Med..

[14] Shode F.O., Idowu A.S.K., Uhomoibhi O.J., Sabiu S. (2021). Repurposing drugs and identification of inhibitors of integral proteins (spike protein and main protease) of SARS-CoV-2. J. Biomol. Struct. Dyn..

[15] Aribisala J.O., Abdulsalam R.A., Dweba Y., Madonsela K., Sabiu S. (2022). Identification of secondary metabolites from Crescentia cujete as promising antibacterial therapeutics targeting type 2A topoisomerases through molecular dynamics simulation. Comput. Biol. Med..

[16] Delijewski M., Haneczok J. (2021). AI drug discovery screening for COVID-19 reveals zafirlukast as a repurposing candidate. Med. Drug Discov..

[17] Farhat N., Khan A.U. (2021). Repurposing drug molecule against SARS-Cov-2 (COVID-19) through molecular docking and dynamics: A quick approach to pick FDA-approved drugs. J. Mol. Model..

[18] Pettersen E.F., Goddard T.D., Huang C.C., Couch G.S., Greenblatt D.M., Meng E.C., Ferrin T.E. (2004). UCSF Chimera—A visualization system for exploratory research and analysis. J. Comput. Chem..

[19] Sabiu S., Balogun F.O., Amoo S.O. (2021). Phenolics profiling of Carpobrotus edulis (L.) N.E.Br. and insights into molecular dynamics of their significance in type 2 diabetes therapy and Its retinopathy complication. Molecules.

[20] BIOVIA, Dassault Systèmes (2021). Discovery Studio, version 21.1.0.

[21] Wang Q., Zhang Y., Wu L., Niu S., Song C., Zhang Z., Lu G., Qiao C., Hu Y., Yuen K.-Y. (2020). Structural and functional basis of SARS-CoV-2 entry by using human ACE2. Cell.

[22] Mannar D., Saville J.W., Zhu X., Srivastava S.S., Berezuk A.M., Tuttle K.S., Marquez A.C., Sekirov I., Subramaniam S. (2022). SARS-CoV-2 Omicron variant: Antibody evasion and cryo-EM structure of spike protein–ACE2 complex. Sciences.

[23] Aribisala J.O., Sabiu S. (2022). Cheminformatics Identification of Phenolics as Modulators of Penicillin-Binding Protein 2a of *Staphylococcus aureus*: A Structure–Activity-Relationship-Based Study. Pharmaceutics.

[24] Acharya A., Agarwal R., Baker M.B., Baudry J., Bhowmik D., Boehm S., Byler K.G., Chen S.Y., Coates L., Cooper C.J. (2020). Supercomputer-Based Ensemble Docking Drug Discovery Pipeline with Application to Covid-19. Chem. Inf. Model..

[25] Uhomoibhi J.O., Shode F.O., Idowu K.A., Sabiu S. (2022). Molecular modelling identification of phytocompounds from selected African botanicals as promising therapeutics against druggable human host cell targets of SARS-CoV-2. J. Mol. Graph. Model..

[26] Ramirex D., Caballero J. (2016). Is it reliable to use common molecular docking methods for comparing the binding affinities of Enantiomer pairs for their protein target?. Int. J. Mol. Sci..

[27] Nasution F., Toepak M.A., Alkaff E.P., Tambunan U.S.F. (2018). Flexible docking-based molecular dynamics simulation of natural product compounds and Ebola virus Nucleocapsid (EBOV NP): A computational approach to discover new drug for combating Ebola. BMC Bioinform..

[28] Du X., Li Y., Xia Y.L., Ai X.M., Liang J., Sang P., Ji X.L., Liu S.Q. (2016). Insights into Protein-Ligand Interactions: Mechanisms, Models, and Methods. Int. J. Mol. Sci..

[29] Kufareva I., Abagyan R. (2012). Methods of protein structure comparison. Methods Mol. Biol..

[30] Lobanov M.Y., Bogatyreva N.S., Galzitskaya O.V. (2008). Radius of gyration as an indicator of protein structure compactness. Mol. Biol..

[31] Fahad M., Al-Khodairy M., Kalim A., Khan M.K., Manogaran S.P., Salman A., Jamal M.A. (2013). In Silico Prediction of Mechanism of Erysolin-induced Apoptosis in Human Breast Cancer Cell Lines, American. J. Bioinform..

[32] Mousavi S.S., Karami A., Haghighi T.M., Tumilaar S.G., Fatimawali I.R., Mahmud S., Celik I.A., Agagündüz D., Tallei T.E., Emran T.B. (2021). In Silico Evaluation of Iranian Medicinal Plant Phytoconstituents as Inhibitors against Main Protease and the Receptor-Binding Domain of SARS-CoV-2. Molecules.

[33] Izadi H., Stewart K.M.E., Penlidis A. (2014). Role of contact electrification and electrostatic interactions in gecko adhesion. J. R. Soc. Interface.

[34] Kretschmer R., Kinzel D., Gonza´ Lez L. (2010). The Role of Hydrogen Bonds in Protein—Ligand Interactions. DFT Calculations in 1,3-Dihydrobenzimidazole-2 Thione Derivatives with Glycinamide as Model HIV RT Inhibitors. Int. J. Quantum Chem..

[35] Yamashita F., Hashida M. (2004). In silico approaches for predicting ADME properties of drugs. Drug Metab. Pharmacokinet..

[36] Lipinski C.A., Lombardo F., Dominy B.W., Feeney P.J. (2001). Experimental and computational approaches to estimate solubility and permeability in drug discovery and development settings. Adv. Drug Deliv. Rev..

[37] Remko M., Boháč A., Kováčiková L. (2011). Molecular structure, p K a, lipophilicity, solubility, absorption, polar surface area, and blood brain barrier penetration of some antiangiogenic agents. Struct. Chem..

[38] Khumbulani M., Alayande K.A., Sabiu S. (2022). Orientin Enhances Colistin-Mediated Bacterial Lethality through Oxidative Stress Involvement". Evid. Based Complement. Altern. Med..

[39] Kitchen D.B., Decornez H., Furr J.R., Bajorath J. (2009). Docking and scoring in virtual screening for drug discovery: Methods and applications. Nat. Rev. Drug Discov..

[40] Bissantz C., Kuhn B., Stahl M.A. (2010). Medicinal chemist’s guide to molecular interactions. J. Med. Chem..

[41] Stella L., Melchionna S. (1998). Equilibration and sampling in molecular dynamics simulations of biomolecules. Chem. Phys..

[42] Al-Karmalawy A.A., Dahab M.A., Metwaly A.M., Elhady S.S., Elkaeed E.B., Eissa I.H., Darwish K.M. (2021). Molecular Docking and Dynamics Simulation Revealed the Potential Inhibitory Activity of ACEIs Against SARS-CoV-2 Targeting the hACE2 Receptor. Front. Chem..

[43] Dalke A. (2013). The FPS fingerprint format and chemfp toolkit. J. Cheminform..

[44] Verma A.K., Ahmed S.F., Hossain M.S., Bhojiya A.A., Mathur A., Upadhyay S.K., Srivastava A.K., Vishvakarma N.K., Barik M., Rahaman M.M. (2021). Molecular docking and simulation studies of flavonoid compounds against PBP-2a of methicillin-resistant. Staphylococcus Aureus J. Biomol. Struct. Dyn..

