# Supplementary material for: Cheminformatics Bioprospection of Broad Spectrum Plant Secondary Metabolites Targeting the Spike Proteins of Omicron Variant and Wild-Type SARS-CoV-2
Source: Metabolites. 2022 Oct 17;12(10):982. doi: 10.3390/metabo12100982 (PMC9611735; doi:10.3390/metabo12100982)
Supplement: Supplementary file 1 [file metabolites-12-00982-s001.zip › metabolites-1955065-supplementary.pdf]

Table S1: Docking scores of 73 LOCM compounds against spike protein of SC-2WT and its omicron variant

Ligands	Sar cov2 Spike protein [Binding affinity (Kcal/mol)]	Omicron variant spike protein [Binding affinity (Kcal/mol)]
1. Kaempferol-7-glucoside_CID_10095180	-7.3	-6.9
2. Neophytadiene_CID_10446	-5.3	-4.2
3. Pyrazole_CID_1048	-3.1	-3.1
4. Isoorientin_CID_114776	-7.2	-7
5. Uracil_CID_1174	-4.4	-4.2
6. Globulol_CID_12304985	-5.5	-6
7. Cyanidin_CID_128861	-6.7	-6.3
8. P-hydroxybenzoic acid_CID_135	-4.9	-5.2
9. Butyl-2-nitropropanoate_CID_13781495	-4.4	-4.2
10. 2,5-dimethyloxazolidine_CID_15326616	-3.8	-3.5
11. Solucritin_CID_160505	-5.6	-5.8
12. Chlorogenic acid _CID_1794427	-7.1	-6.1
13. 5-dimethyl-4-hydroxy-3 (2H)-furanone3D_CID_19309	-4.2	-4.4
14. Benzene propanoic acid_CID_20569239	-5.4	-5.2
15. Helenalin_CID_23205	-5.7	-6.6
16. Bastadin_CID_23426999	-8.1	-7.7
17. 5-hydroxymethylfurfural_CID_237332	-4.6	-4.5
18. 4-mercaptophenol3D_CID_240147	-4.3	-4.3
19. Benzoic acid_CID_243	-4.6	-5.3
20. Aureonitol_CID_25064137	-5.6	-4.8
21. 1-Tridecanal_CID_25311	-4.9	-4.1
22. Hexadecanol_CID_2682	-4.9	-3.9
23. 1,2.4,5-Tetrazine-3,6-diamine_CID_283379	-4.3	-4.5
24. Phosphonous acid_CID_3014827	-2.4	-2.5
25. eugenol_CID_3314	-5.2	-5.3
26. Xylocaine_CID_3676	-5	-5.4
27. 2-Galactopyranose_CID_439804	-4.7	-5
28. Pelargonidin_CID_440832	-6.3	-6.4
29. Luteolinidin_CID_441701	-6.8	-6.3
30. 6-Hydroxylcyanidin-3-3D_CID_44257027	-7.2	-7.2
31. Cinnamic acid_CID_444539	-5.2	-5.4
32. farnesol_CID_445070	-6.1	-4.8
33. Cyanindin-3-glucoside_CID_4481259	-6.6	-6.6
34. kaur-16-ene_CID_520687	-6.4	-7.2
35. 1,3,5-Triazine-2,4,6-triamine3D_CID_5250297	-4.5	-4.8
36. Phytol3D_CID_5280435	-5.4	-4.5
37. Apigenin _CID_5280443	-6.6	-6.7
38. Luteolin_CID_5280445	-6.7	-6.4
39. Cistanoside C_CID_5315929	-7.1	-6.7
40. Cistanoside D_CID_5315930	-6.8	-6.3
41. Geraniin_CID_5317050	-7.1	-7.5
42. 9,17-octadecadienal_CID_5365667	-5.2	-4
43. 2,5-difluorophenylhydrazine_CID_588957	-4.9	-5.2
44. D-Galactopyranose_CID_6036	-4.7	-5
45. 1,2-ethenediamine_CID_6066279	-2.5	-2.6
46. 3,5-dimethyl-1H-pyrazole_CID_6210	-4	-4
47. 2-Hydroxycinnamic acid_CID_637540	-5.2	-5.5
48. Hydroxycinnamic acid_CID_637542	-5.1	-5.6
49. cis-sabinene hydrate_CID_6427493	-5.2	-5.4

50. Trans-pinocamphone_CID_6430551	-5.1	-5.2
51. Epigallocatechin gallate_CID_65064	-6.9	-7.2
52. Acrylic acid_CID_6581	-3.4	-3.1
53. Pinocembrin_CID_68071	-6.2	-6.9
54. Hordenine_CID_68313	-4.8	-5
55. Thymol_CID_6989	-5.2	-5.2
56. 2-methyl-napthalene3D_CID_7055	-5.6	-6.2
57. Maysin_CID_70698181	-8.4	-7.5
58. Isoflavone_CID_72304	-6.6	-6.2
59. 3-pentanone_CID_7288	-3.4	-3.3
60. Furfural_CID_7362	-4	-3.5
61. citronellol_CID_7793	-4.7	-4.2
62. 4H-pyran-4-one_CID_7968	-3.6	-3.4
63. Isopropyl myristate_CID_8042	-5.2	-4.3
64. O-diethyl phthalate_CID_8554	-4.9	-5.4
65. Hexanoic acid_CID_8892	-4.3	-4
66. T-butylthiothioacetic acid_CID_90460	-4.1	-3.9
67. Catalpol_CID_91520	-6.3	-6.5
68. Indolizine_CID_9230	-4.5	-4.8
69. Ledol_CID_92812	-5.6	-6.3
70. Catalposide_CID_93039	-7.4	-6.8
71. Naringenin_CID_932	-6.3	-6.7
72. Palmitic acid_CID_985	-5.3	-4.2
73. Phenol_CID_996	-4.3	-4.4
Controls		
Zafirlukast	-7.9	-7.4
Cefoperazone	-6.7	-6.2

Table S2: 2D interaction plots of top five LOCM compounds against SP of omicron and SC-2WT after 100 ns simulation

Complex	2D interactions
Omicron	
6-Hydroxycyanidin 3-rutinoside	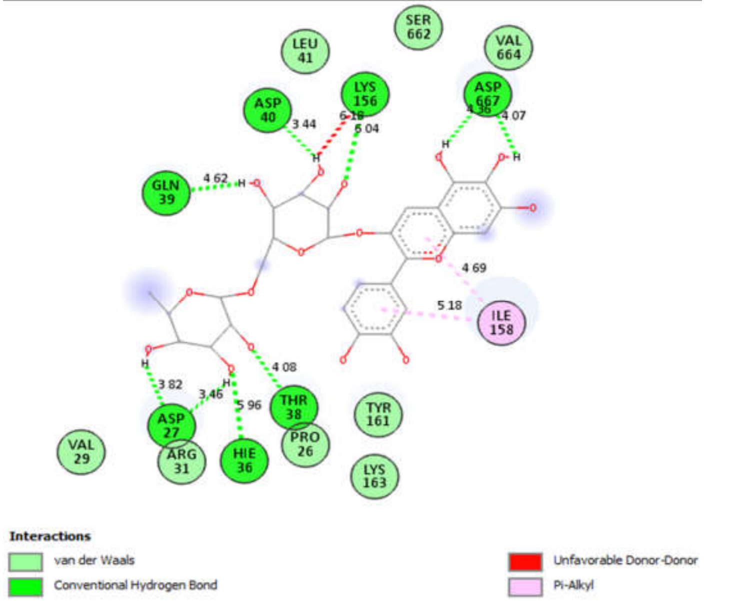

Epigallocatechin gallate	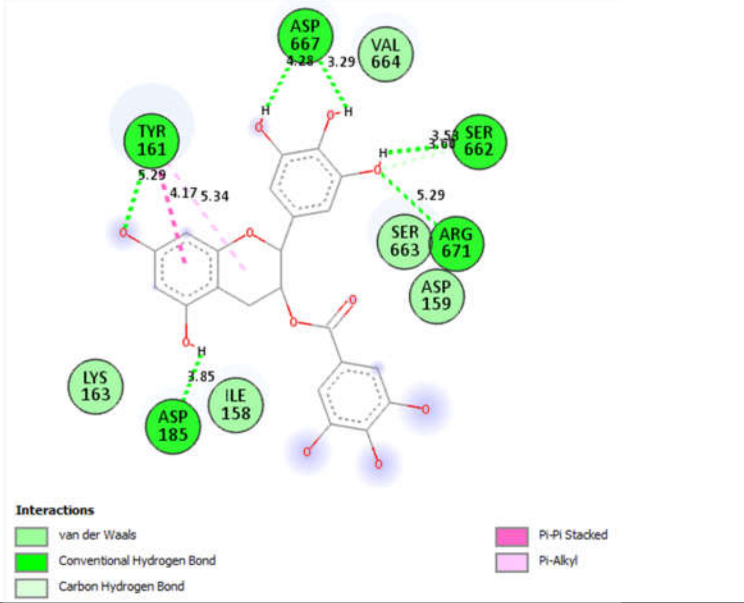
Geraniin	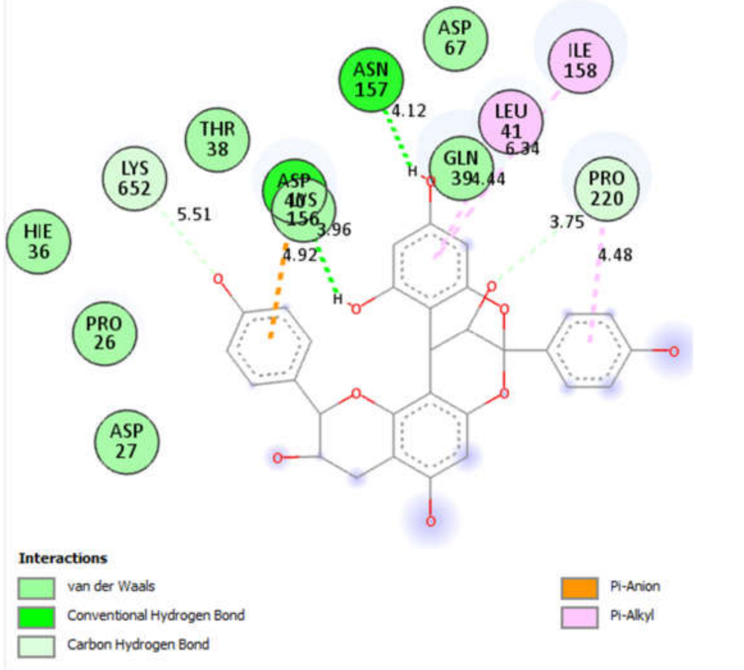

Kaempferol-7-glucoside	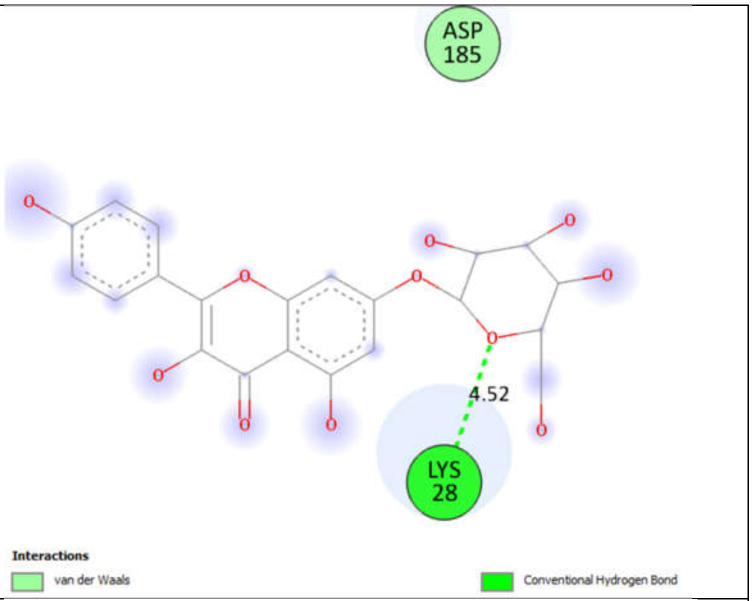
Maysin	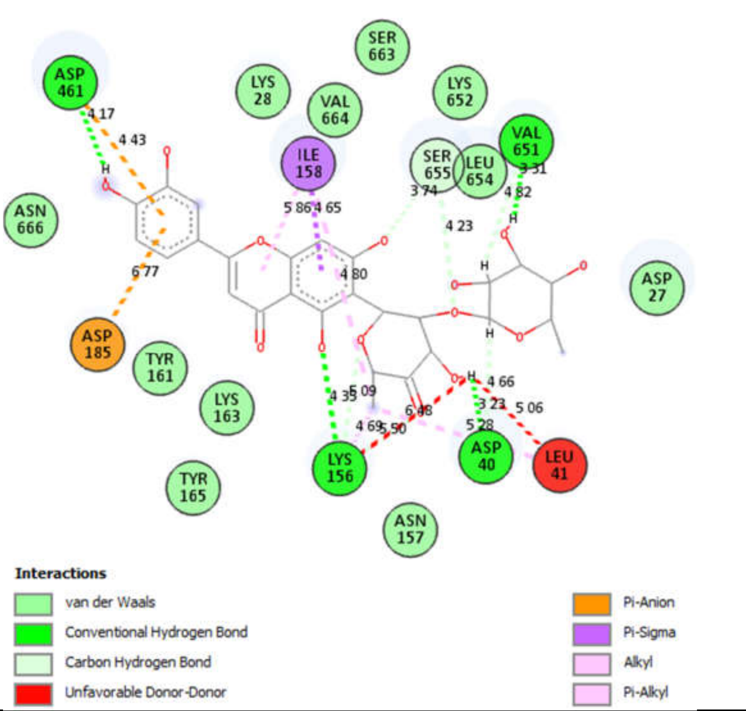

<table>
<tr><td>Zafirlukast</td><td>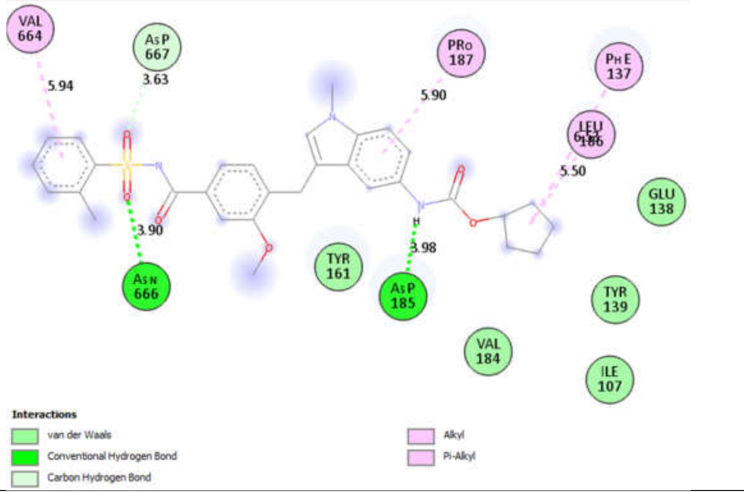
</td></tr>
<tr><td colspan="2">SARS-CoV-2</td></tr>
<tr><td>6-Hydroxycyanidin 3-rutinoside</td><td>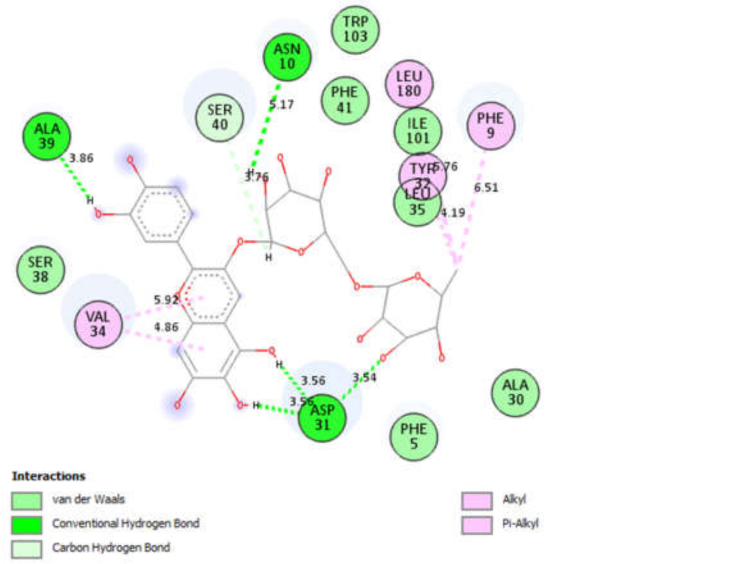
</td></tr>
<tr><td>Catalposide</td><td>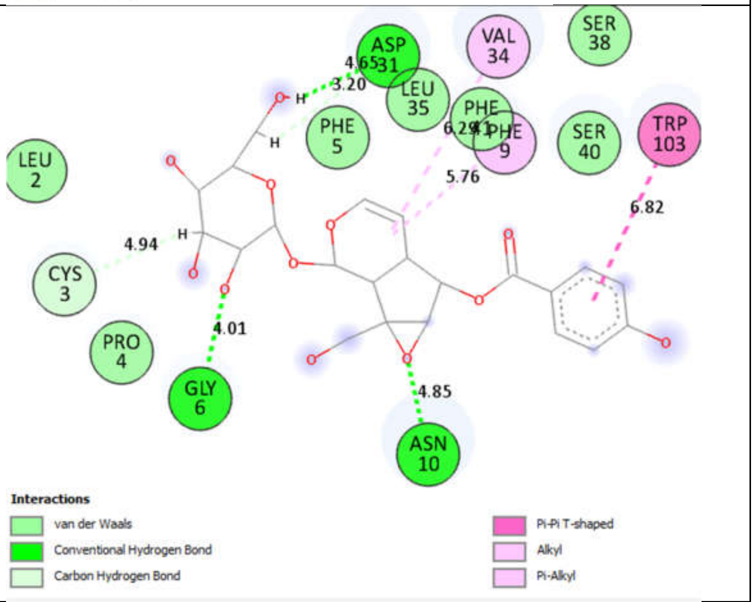
</td></tr>
</table>

Geraniin	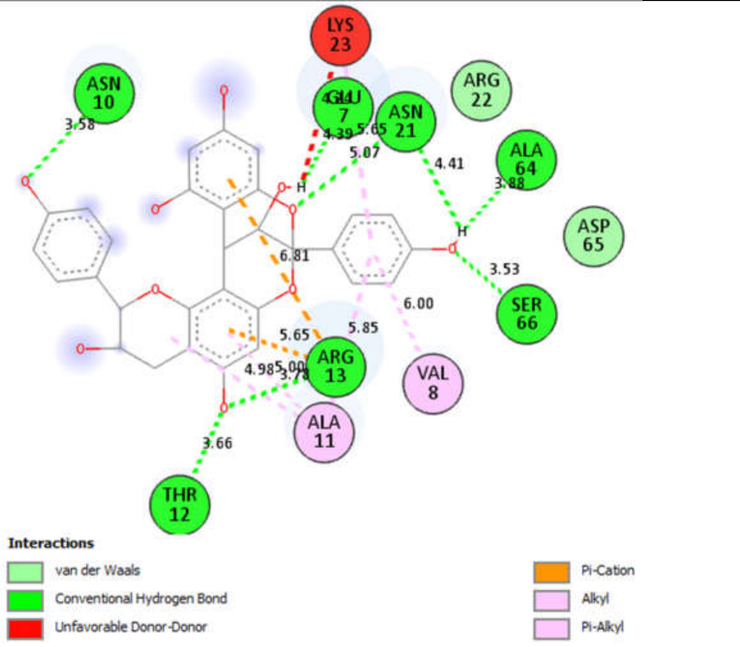
Kaempferol-7-glucoside	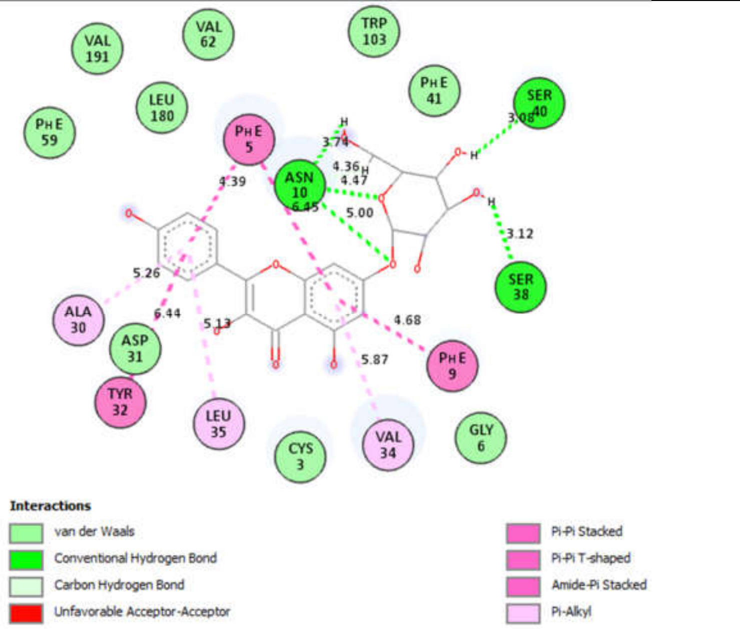

Maysin	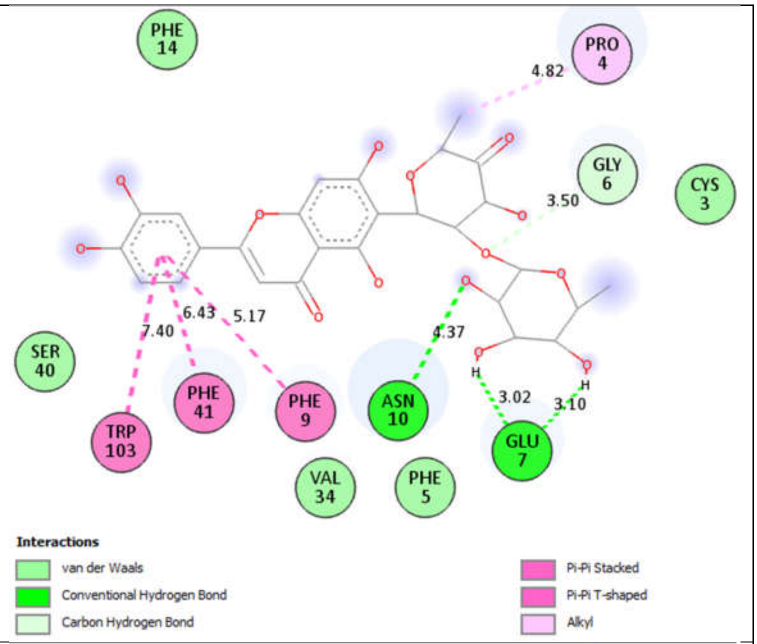
Zafirlukast	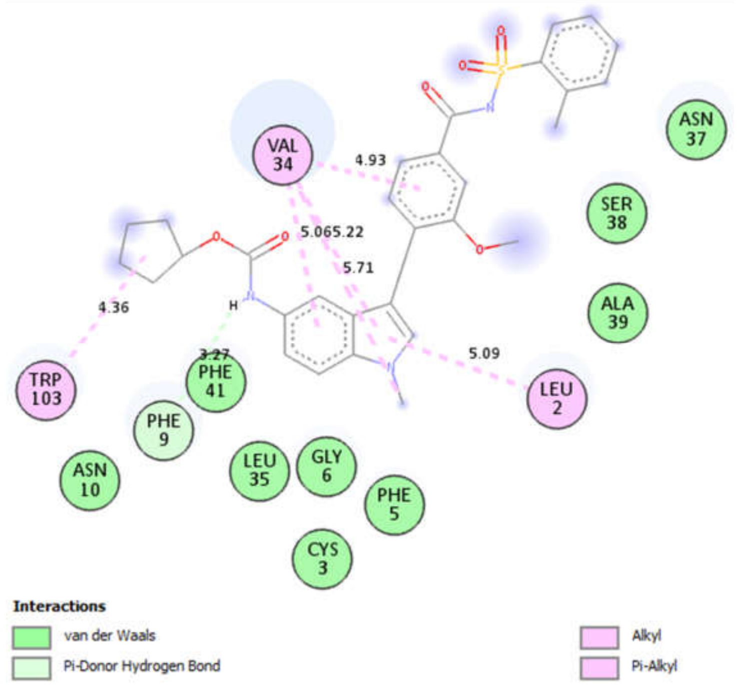
